# Characteristic Metabolic Changes in Skeletal Muscle Due to Vibrio vulnificus Infection in a Wound Infection Model

**DOI:** 10.1128/msystems.00682-22

**Published:** 2023-03-20

**Authors:** Kai Ishida, Takaaki Shimohata, Yuna Kanda, Anh Quoc Nguyen, Rumiko Masuda, Kohei Yamazaki, Takashi Uebanso, Kazuaki Mawatari, Takashige Kashimoto, Akira Takahashi

**Affiliations:** a Department of Preventive Environment and Nutrition, Institute of Biomedical Sciences, Tokushima University Graduate School, Tokushima, Japan; b Department of Microbial Control, Institute of Biomedical Sciences, Tokushima University Graduate School, Tokushima, Japan; c Faculty of Marine Biosciences, Fukui Prefectural University, Fukui, Japan; d Department of Food Microbiology and Molecular Biology, National Institute of Nutrition, Hanoi, Vietnam; e Department of Clinical Nutrition and Food Management, Institute of Biomedical Sciences, Tokushima University Graduate School, Tokushima, Japan; f Laboratory of Veterinary Public Health, School of Veterinary Medicine, Kitasato University, Towada, Aomori, Japan; Chan Zuckerberg Biohub

**Keywords:** CE-TOFMS, host metabolomics, MARTX toxin, TCA cycle, *Vibrio vulnificus*

## Abstract

Vibrio vulnificus is a bacterium that inhabits warm seawater or brackish water environments and causes foodborne diseases and wound infections. In severe cases, V. vulnificus invades the skeletal muscle tissue, where bacterial proliferation leads to septicemia and necrotizing fasciitis with high mortality. Despite this characteristic, information on metabolic changes in tissue infected with V. vulnificus is not available. Here, we elucidated the metabolic changes in V. vulnificus-infected mouse skeletal muscle using capillary electrophoresis time-of-flight mass spectrometry (CE-TOFMS). Metabolome analysis revealed changes in muscle catabolites and energy metabolites during V. vulnificus infection. In particular, succinic acid accumulated but fumaric acid decreased in the infected muscle. However, the virulence factor deletion mutant revealed that changes in metabolites and bacterial proliferation were abolished in skeletal muscle infected with a multifunctional-autoprocessing repeats-in-toxin (MARTX) mutant. On the other hand, mice that were immunosuppressed via cyclophosphamide (CPA) treatment exhibited a similar level of bacterial counts and metabolites between the wild type and MARTX mutant. Therefore, our data indicate that V. vulnificus induces metabolic changes in mouse skeletal muscle and proliferates by using the MARTX toxin to evade the host immune system. This study indicates a new correlation between V. vulnificus infections and metabolic changes that lead to severe reactions or damage to host skeletal muscle.

**IMPORTANCE** V. vulnificus causes necrotizing skin and soft tissue infections (NSSTIs) in severe cases, with high mortality and sign of rapid deterioration. Despite the severity of the infection, the dysfunction of the host metabolism in skeletal muscle triggered by V. vulnificus is poorly understood. In this study, by using a mouse wound infection model, we revealed characteristic changes in muscle catabolism and energy metabolism in skeletal muscle associated with bacterial proliferation in the infected tissues. Understanding such metabolic changes in V. vulnificus-infected tissue may provide crucial information to identify the mechanism via which V. vulnificus induces severe infections. Moreover, our metabolite data may be useful for the recognition, identification, or detection of V. vulnificus infections in clinical studies.

## INTRODUCTION

Vibrio vulnificus is a halophilic Gram-negative bacterium that inhabits warm seawater or brackish water environments. V. vulnificus causes foodborne disease after the ingestion of raw or undercooked seafood, characterized by several gastrointestinal symptoms, such as abdominal pain, vomiting, fever, chills, and nausea, which resolve within a few days in healthy people (1–3). Also, this bacterium causes wound infection via contaminated seawater (4, 5). In severe cases, the invasion of bacteria into skeletal muscle is commonly found not only in wound infection but also in oral infection (6, 7). Moreover, patients with underlying diseases, such as chronic liver disease, diabetes, hemochromatosis, immunocompromised states, or serum iron-elevating conditions, may develop a severe infection that leads to septicemia and necrotizing manifestation (3, 8). V. vulnificus-induced acute septicemia entails a high mortality rate, exceeding 50% within 24 to 72 h after infection (9, 10). In addition, acute V. vulnificus infection induces severe rapidly progressive necrotizing fasciitis and tissue necrosis in host skeletal muscle. According to epidemiological studies, V. vulnificus exhibited the highest rate of infection and accounted for 25% of the *Vibrio* species detected in parenteral infections in the United States (1). Another study reported that V. vulnificus causes V. vulnificus necrotizing skin and soft tissue infection (VNSSTIs), and more than 95% of cases of VNSSTIs are associated with the subtropical western Pacific and Atlantic coastal regions of the Northern Hemisphere, such as Taiwan, South Korea, Japan, or the Gulf of Mexico (11).

Vibrio vulnificus hemolysin (VVH) and the multifunctional-autoprocessing repeats-in-toxin (MARTX) are regarded as significant toxins of V. vulnificus. Researchers have identified several functions for each toxin. VVH is a pore-forming toxin that promotes bacterial translocation from the intestine to the bloodstream by causing intestinal tissue damage with cell death (12). VVH exerts several functions in host cells, such as causing permeabilization of the intestinal epithelium in association with apoptosis and cell death, with reactive-oxygen-species (ROS) production (13) or autophagy activation (14). MARTX is commonly conserved in several Gram-negative bacteria, including another *Vibrio* species (15). MARTX is associated with numerous cytopathic and cytotoxic functions in eukaryotic cells, as it causes cytoskeletal dysfunction (16), inflammasome activation (17), inhibition of phagocytosis (18, 19), and induction of apoptosis (20). Several studies have reported that V. vulnificus toxins injured host epithelial cells with the disruption of the host mitochondrial function, and that these damages played a role in the disruption of the central metabolic system in host cells *in vitro* (21, 22). Those studies suggest that V. vulnificus induces the degradation of skeletal muscle and host metabolic changes via the action of its characteristic toxins. Necrotizing fasciitis is a major symptom of V. vulnificus infection that is found in severe cases of infection with dramatic symptoms in skeletal muscle. However, the relationship between necrotizing fasciitis and the metabolic changes that occur during V. vulnificus infection remains unknown.

Omics analyses (genomics, transcriptomics, proteomics, and metabolomics) have been used to investigate the mechanism of necrotizing fasciitis in bacterial infection. In particular, a metabolic analysis indicated differences in metabolites during the acute phase of infection in patients. Moreover, previous studies have indicated that bacterial growth and viability were closely associated and resulted in host metabolic changes. In fact, a variety of group A streptococci (GAS) respond to specific metabolites (23). According to a recent study, metabolic changes in infected tissues of the host may contribute to promoting bacterial growth and/or virulence, which exacerbates host symptoms and physiological conditions (24). However, it is unknown how V. vulnificus triggers metabolic changes in the host. To identify the mechanism of bacterial infection, it is necessary to investigate the metabolic changes that occur in V. vulnificus-infected tissues.

In this study, we attempted to estimate the metabolic changes in V. vulnificus-infected skeletal muscles in a mouse wound infection model using capillary electrophoresis–time-of-flight mass spectrometry (CE-TOFMS). We found that muscle catabolites changed upon infection by *Vibrio* spp., whereas V. vulnificus alone significantly affected the succinate-fumarate pathway of the tricarboxylic acid (TCA) cycle in skeletal muscle. The V. vulnificus toxin MARTX facilitated the proliferation of bacteria in skeletal muscle and was indirectly important for triggering metabolic changes in mouse skeletal muscle. Even during short infection, these metabolic changes were detected in skeletal muscle. This study is the first report showing metabolic changes in V. vulnificus-infected muscle tissue.

## RESULTS

### Catabolites of mouse skeletal muscle were detected in the mouse skeletal muscle and blood after infection with V. vulnificus.

Prior to the investigation of the metabolic changes in the V. vulnificus wound infection model, we checked that V. vulnificus induces inflammation of skeletal muscle (25). After 9 h of infection, V. vulnificus-injected skeletal muscle tissues exhibited edema and neutrophil infiltration (see Fig. S1A in the supplemental material) and exhibited a high transcriptional level of *MIP-2*, *TNF-α*, and *IL-6* (Fig. S1B). These data showed that V. vulnificus induced inflammatory reactions in the injected tissues of our mouse model.

10.1128/msystems.00682-22.1 FIG S1 V. vulnificus caused inflammation in mouse skeletal muscles. Mouse skeletal muscles were treated with PBS or V. vulnificus for 9 h. (A) Hematoxylin and eosin staining showing the effect of PBS treatment or V. vulnificus infection on mouse skeletal muscles. (B) The expression levels of the *MIP-2*, *TNF-α*, and *IL-6* mRNAs were estimated by RT-qPCR. The expression of each mRNA was normalized to that of the *GAPDH* mRNA. These samples were divided into four groups as follows: PBS-treated leg (PBS), V. vulnificus-injected leg (Vv), the leg opposite the PBS-treated leg (OP), and the leg opposite the V. vulnificus-injected leg (OV) (9 or 10/group). (C) Concentrations of arginine, creatine, and creatinine in mouse blood were extracted from the CE-TOFMS data at 9 h after infection. The samples were divided into four groups as described above (3/group). *, *P *< 0.05, and **, *P *< 0.01, versus the control group (*t* test). *P* values were determined by one-way ANOVA followed by Dunnett’s test. ^†^, FDR < 0.05, versus the Vv group. Download FIG S1, TIF file, 8.2 MB. Copyright © 2023 Ishida et al. 2023 Ishida et al. https://creativecommons.org/licenses/by/4.0/ This content is distributed under the terms of the Creative Commons Attribution 4.0 International license.

**FIG 1:**
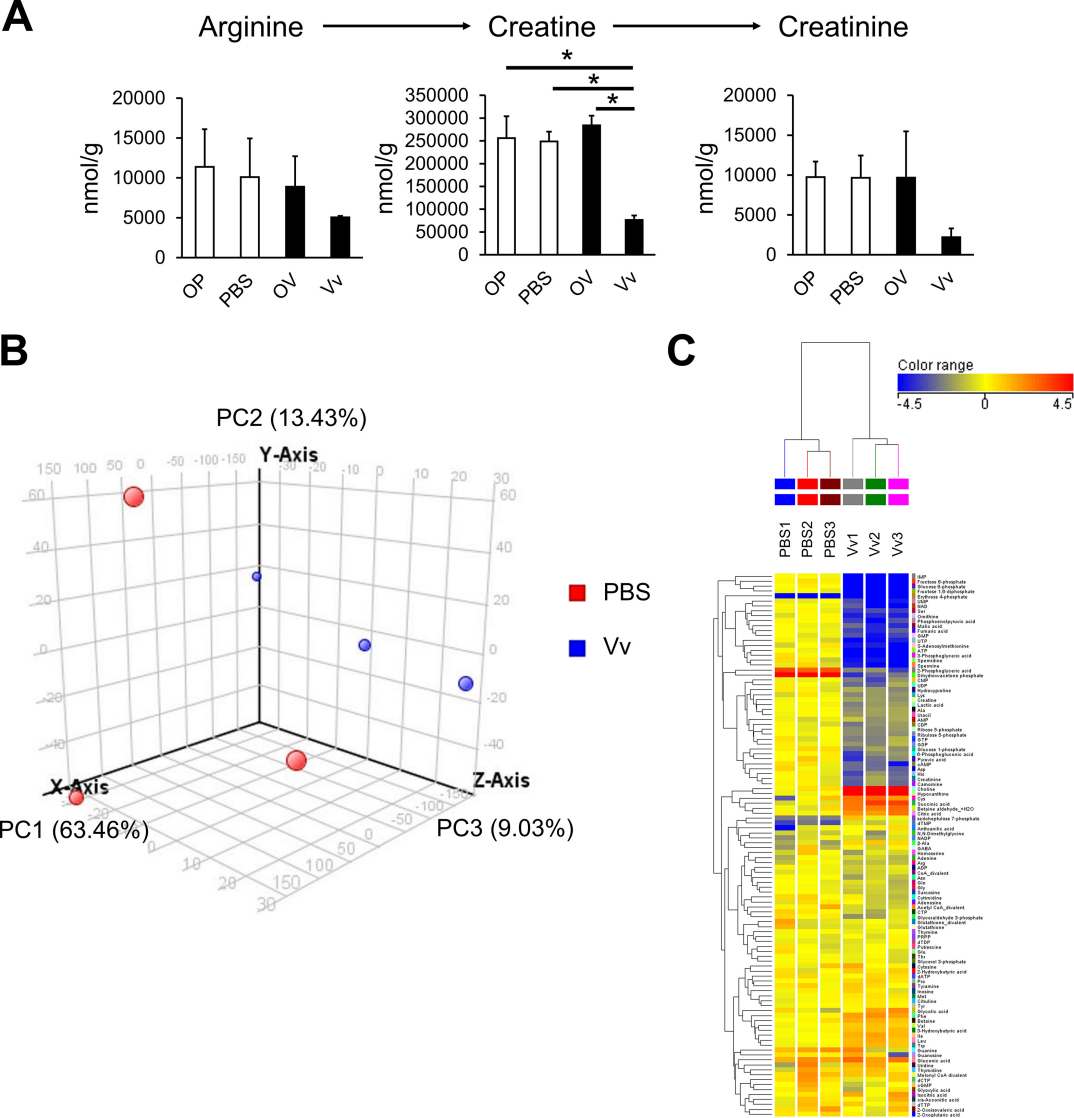

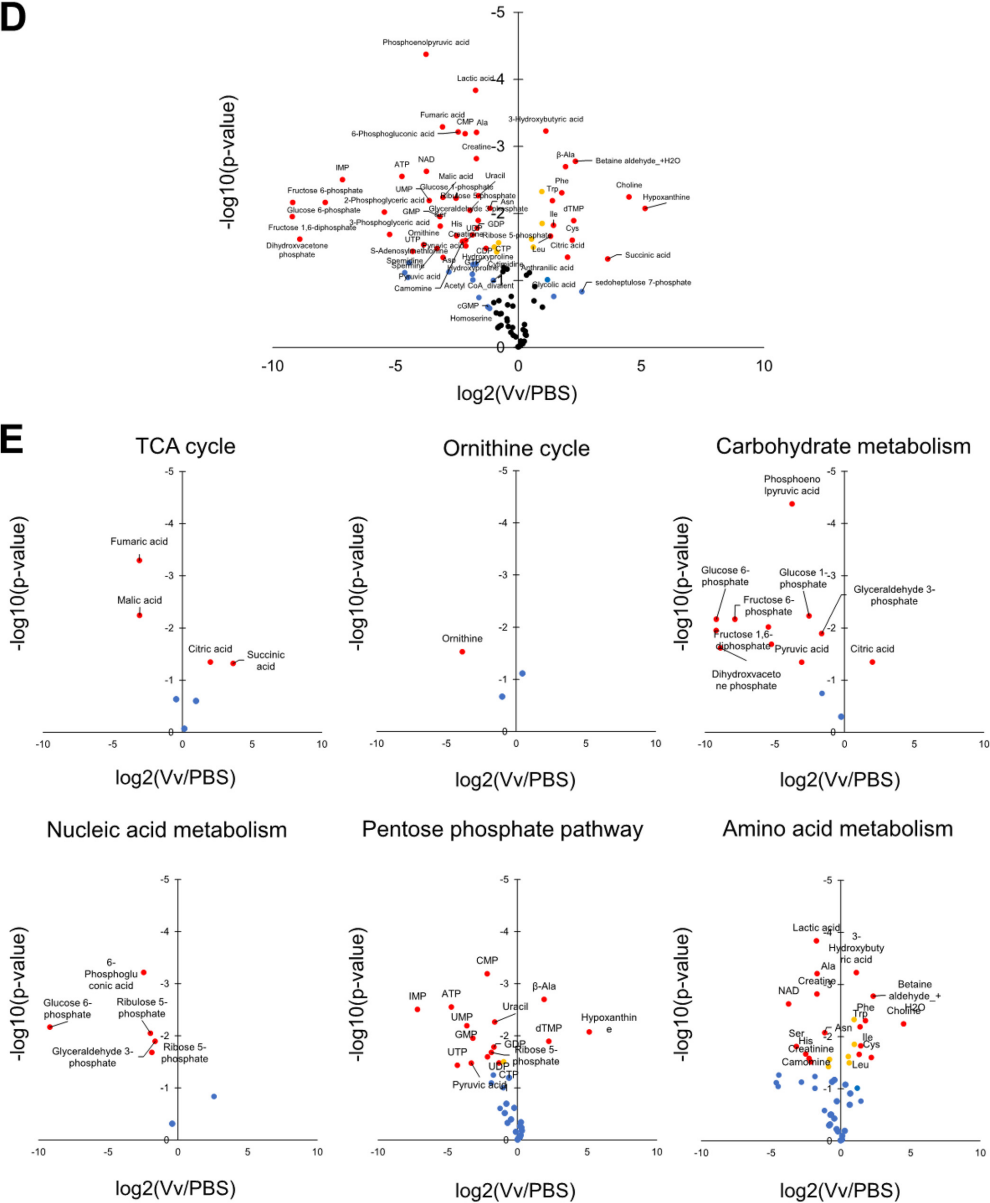
Profiling of the mouse skeletal muscle metabolites triggered by V. vulnificus infection. The metabolite profiles of mouse skeletal muscles 9 h after infection. (A) Concentrations of arginine, creatine, and creatinine metabolites in skeletal muscles were extracted from CE-TOFMS data at 9 h after infection. The samples were divided into four groups: PBS-treated leg (PBS), V. vulnificus-injected leg (Vv), leg opposite the PBS-treated leg (OP), and leg opposite the V. vulnificus-injected leg (OV) (3/group). (B) PCA plots showing different clustering between the PBS and Vv groups. The red and blue dots indicate PBS- and V. vulnificus (Vv)-injected tissue. *x* axis, 63.46%; *y* axis, 13.43%; *z* axis, 9.03% (3/group). (C) Heat map analysis of the metabolic changes to the fold change of each metabolite using the autoscaling method. Red and blue indicate high and low concentrations of metabolites, respectively (3/group). (D) Volcano plot showing relative changes in the metabolites in the Vv group, with red denoting significantly changed metabolites (by more than 2-fold), blue indicating metabolites that changed (by more than 2-fold) but not significantly, and orange indicating significant differences which were less than 2-fold (3/group). (E) Volcano plot showing relative changes of the metabolites in the Vv group, categorized into six metabolic pathways (TCA cycle, carbohydrate metabolism, pentose phosphate pathway, amino acid metabolism, ornithine cycle, and nucleic acid metabolism) (3/group). *P* values were determined by one-way ANOVA followed by Dunnett’s test. *, FDR < 0.05 compared with the Vv group.

In accordance with inflammation of the skeletal muscle, next we measured changes in the metabolite profile of the infection model mice. Creatine has been used as a creatine-phosphokinase-mediated muscle catabolism indicator in general clinical tests (26). Therefore, to estimate the muscle catabolism in V. vulnificus infection, we analyzed the metabolic conversion of ornithine to creatinine in host skeletal muscle after 9 h of V. vulnificus infection. Mouse skeletal muscles were treated by subcutaneous injection into the right femoral region of phosphate-buffered saline (PBS; control) or V. vulnificus (Vv; wild type), and each opposite leg was defined as OP (leg opposite the PBS leg) or OV (leg opposite the V. vulnificus leg). These skeletal muscles were collected after 9 h of infection, and the metabolites were analyzed by CE-TOFMS. We found that creatine and creatinine were decreased in the Vv group (Fig. 1A). Conversely, among the blood metabolites, creatine and creatinine were increased in the Vv group sample (Fig. S1C). In this mouse model, we found an increase of creatine and creatinine in blood similarly to the general clinical test, whereas we found a decrease of creatine and creatinine in V. vulnificus-infected skeletal muscle. These data indicated that V. vulnificus infection causes metabolic changes in skeletal muscle and imply the existence of other uncovered metabolic changes.

### Metabolic changes in the skeletal muscles of the V. vulnificus wound infection mouse model.

Next, we conducted a comprehensive analysis of skeletal muscle metabolites and searched for significantly altered metabolites. A total 110 metabolites were identified (54 and 56 metabolites were identified in the cationic and anionic mode, respectively), using CE-TOFMS (Table S1). These data were analyzed via a principal-component analysis (PCA) and a hierarchical clustering heat map analysis. The PCA plots indicated a clear metabolite difference between the metabolites of the PBS and Vv groups (Fig. 1B). The heat map data showed characteristic metabolite patterns in the PBS and Vv groups (Fig. 1C). These data showed that V. vulnificus infection induced apparent metabolic changes. Furthermore, a volcano plot showed that there were 13 increased metabolites and 36 decreased metabolites in V. vulnificus-infected skeletal muscle compared with those of PBS-treated muscle (Fig. 1D). The data show the relative values of each metabolite identified by CE-TOFMS in V. vulnificus-infected mice. The detected metabolites are classified into six metabolic pathways (tricarboxylic acid [TCA] cycle, carbohydrate metabolism, pentose phosphate pathway, amino acid metabolism, ornithine cycle, and nucleic acid metabolism). Those data indicated changes in metabolite associated with infection damage (Fig. 1E). Upon analysis of each metabolic pathway, the amino acid levels in infected tissues were elevated, which suggests the degradation of proteins in infected tissues. Analysis of catabolism in tissues infected with V. vulnificus showed that most metabolites, such as nucleic acids, pentose phosphate, and carbohydrates, were decreased. Conversely, the metabolites of the TCA cycle were specifically increased and decreased. In this experiment, we also analyzed the metabolites in blood samples from PBS-treated mice and the V. vulnificus-infected mouse wound infection model (Table S1). In contrast with the muscle samples, we could not identify a characteristic difference in blood samples using PCA (Fig. S2A), the heat map data (Fig. S2B), or the volcano plot (Fig. S2C). Furthermore, most metabolic pathways were not changed in the V. vulnificus-infected group relative to the PBS-treated group (Fig. S2D). These data indicate that V. vulnificus affected the host metabolism and that its metabolic changes were specifically induced at the site of infection in our mouse wound infection model.

10.1128/msystems.00682-22.2 FIG S2 Profiling of the mouse blood metabolites triggered by infection with V. vulnificus. Metabolites in mouse whole blood were measured by CE-TOFMS after 9 h of infection with V. vulnificus. (A) PCA plots showing configure to different cluster between PBS- and Vv-treated tissues. The red and blue dots indicate PBS and V. vulnificus (Vv)-infected tissues (3 to 5/group). (B) A heat map analysis reported the metabolic changes of each metabolite via the autoscaling method; the results are expressed using a red and blue color scheme, indicating high and low concentrations of metabolites, respectively. *x* axis, 25.67%; *y* axis, 16.99%; *z* axis, 18.06% (3 to 5/group). (C) The relative changes in the metabolites in the Vv group are indicated by the volcano plot, with red denoting significantly changed metabolites (by more than 2-fold), blue indicates metabolites that changed by more than 2-fold but without significant differences detected, and orange indicates metabolites with significant differences but showing changes of less than 2-fold (3 to 5/group). (D) The relative changes of the metabolites in the Vv group were indicated by the volcano plot in Vv-infected mice were categorized into six metabolic pathways (TCA cycle, carbohydrate metabolism, pentose phosphate pathway, amino acid metabolism, ornithine cycle, and nucleic acid metabolism) (3 to 5/group). Download FIG S2, TIF file, 0.6 MB. Copyright © 2023 Ishida et al. 2023 Ishida et al. https://creativecommons.org/licenses/by/4.0/ This content is distributed under the terms of the Creative Commons Attribution 4.0 International license.

10.1128/msystems.00682-22.5 TABLE S1 Calculated concentrations of metabolites in this study. Download Table S1, PDF file, 0.3 MB. Copyright © 2023 Ishida et al. 2023 Ishida et al. https://creativecommons.org/licenses/by/4.0/ This content is distributed under the terms of the Creative Commons Attribution 4.0 International license.

### Changes to the TCA cycle caused by V. vulnificus infection.

**FIG 2:**
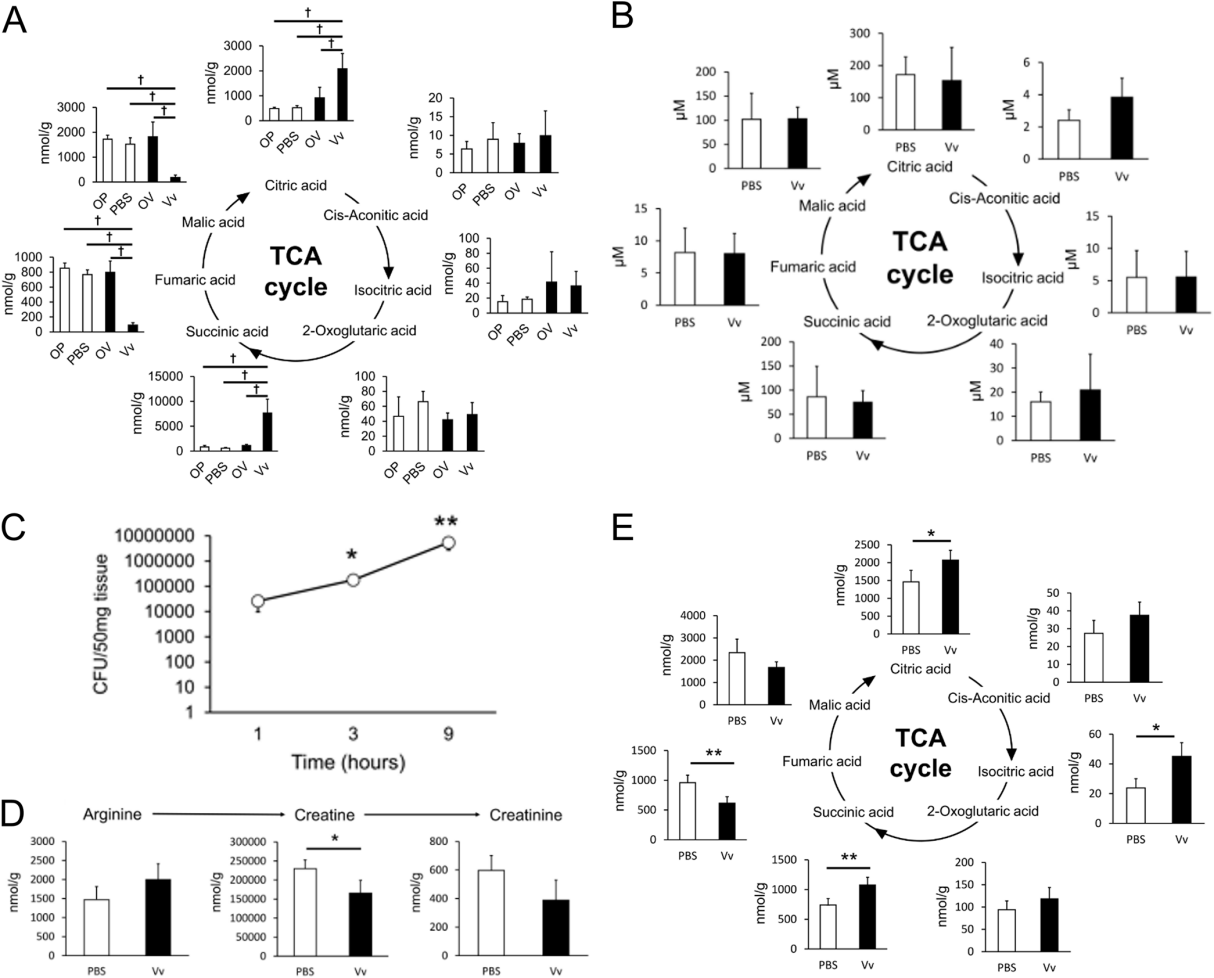
Changes in the metabolic pathway of energy synthesis in mouse skeletal muscles compared with blood or early infected samples. (A) Concentration of the TCA cycle metabolites in skeletal muscles at 9 h after infection. The samples were divided into four groups, as follows: PBS-treated leg (PBS), V. vulnificus-injected leg (Vv), leg opposite the PBS-treated leg (OP), and leg opposite the V. vulnificus-injected leg (OV) (3/group). (B) Concentrations of the TCA cycle metabolites in whole blood were extracted from CE-TOFMS data at 9 h after infection (3 to 5/group). (C) The number of bacteria in skeletal muscle tissues was estimated using the CFU assay. The bacteria were collected in skeletal muscles after 1, 3, and 9 h of infection by V. vulnificus (4/group). (D) Metabolites of the arginine-to-creatinine pathway in skeletal muscles were extracted from CE-TOFMS data at 3 h after infection (4/group). (E) Metabolites of the TCA cycle in skeletal muscles were extracted from CE-TOFMS data at 3 h after infection (4/group). Each bar indicates the mean concentration. *, *P *< 0.05, and **, *P *< 0.01, versus the control group (by *t* test). One-way ANOVA with Tukey’s test was used to determine the differences in each group. *P* values were determined by one-way ANOVA followed by Dunnett’s test. ^†^, FDR < 0.05, versus the Vv group.

The TCA cycle is a central pathway for oxidative phosphorylation that works in the generation of energy. These enzymes are localized in mitochondria, which are organelles that are often injured by bacterial infections. In fact, P. aeruginosa modulates the host TCA cycle in a variety of tissues (27). To capture the metabolic changes of the TCA cycle in V. vulnificus infection, we measured the organic acids in the TCA cycle (i.e., citric acid, *cis*-aconitic acid, isocitric acid, 2-oxoglutaric acid, succinic acid, fumaric acid and malic acid) (Fig. 2A and B). In V. vulnificus-infected muscle, the citric acid and succinic acid was increased, whereas fumaric acid and malic acid were decreased. Interestingly, the pattern of metabolic change of succinic acid was completely opposite to that of fumaric acid. In contrast to V. vulnificus-infected muscle, metabolic change patterns were not found in blood. These data indicated that V. vulnificus caused metabolic changes in the TCA cycle in an infection site-specific manner.

In order to reveal the metabolic change in the early infection phase, we investigated the bacterial cell counts between the different infection time points in the host skeletal muscle and measured metabolites in the host skeletal muscle after 3 h of infection (Fig. 2C to E). The bacterial cell counts in infected skeletal muscle were dramatically increased after 9 h of infection. Moreover, even at 3 h of infection, our data indicated the presence of changes in muscle catabolism and TCA cycle metabolites in V. vulnificus-infected skeletal muscle. These data imply that the muscle catabolism and TCA cycle metabolites are associated with bacterial growth.

### Association between V. vulnificus secreted toxins and metabolic changes in mouse skeletal muscle.

**FIG 3:**
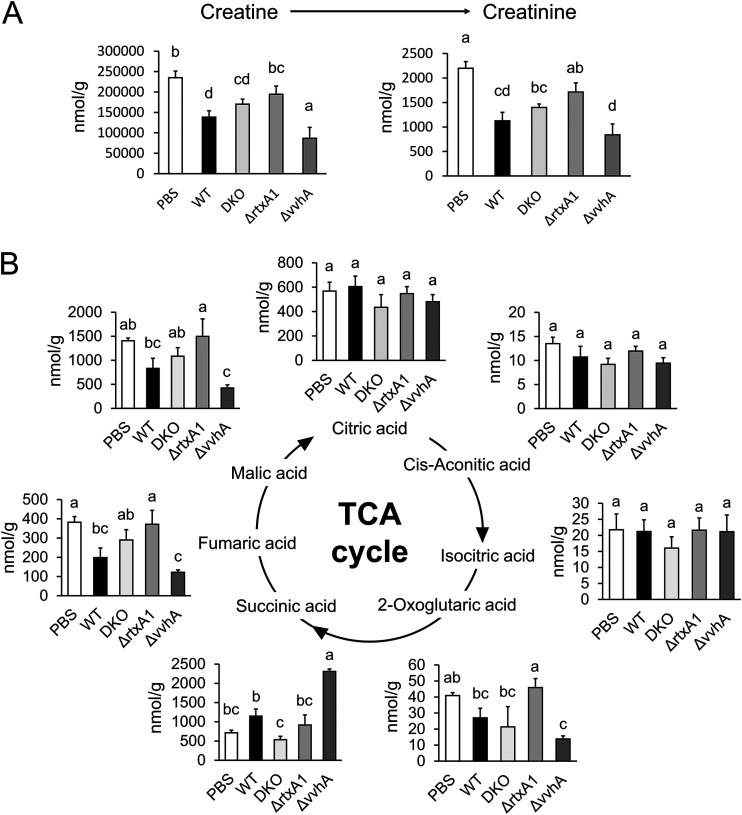
The *rtxA1* deletion mutant strain abolished the V. vulnificus-triggered metabolic changes in mouse skeletal muscle. (A) Concentrations of the creatine and creatinine metabolites in skeletal muscles were extracted from the CE-TOFMS data after treatment with PBS or infection with the V. vulnificus strains (WT, DKO, Δ*rtxA1*, and Δ*vvhA* strains) for 9 h (4/group). (B) Concentration of the TCA cycle metabolites in skeletal muscles were extracted from the CE-TOFMS data after treatment with PBS or infection with the V. vulnificus strains (WT, DKO, Δ*rtxA1*, and Δ*vvhA* strains) for 9 h (4/group). Each bar indicates the mean concentration. *P* values were determined by one-way ANOVA followed by Tukey’s test. Different letters indicate significant differences (*P* < 0.05).

Previous studies revealed that VVH and MARTX induced apoptosis and mitochondrial damage (20, 21). To estimate the effect of the toxins on the metabolic changes of the TCA cycle, we used mutant strains with single and double deletions of the VVH and MARTX toxins (the Δ*vvhA* and Δ*rtxA1* mutants and the double knockout [DKO] mutant) in the V. vulnificus wound infection model. First, we confirmed the inflammation of the skeletal muscle in the presence of V. vulnificus toxins (Fig. 3A and B). The *rtxA1* deletion mutant and the DKO strain exhibited a decrease in bacterial survival and proliferation in skeletal muscle and a decrease in inflammation in skeletal muscle (Fig. S3A and B). Taken together, these data confirm that MARTX contributes to inflammation in the skeletal muscle of V. vulnificus infection. The *rtxA1* mutant strain and double deletion strain (Δ*rtxA1* and DKO) showed a decrease in metabolic changes in the creatine to creatinine pathway in skeletal muscle. As shown in Fig. 3B, the *vvhA* deletion mutant exhibited metabolic changes in the TCA cycle metabolites in host skeletal muscle that were similar to those observed under wild-type conditions. However, these metabolic changes were abolished in the *rtxA1* deletion mutant. These data indicate that MARTX is associated with the modulation of the host TCA cycle and skeletal muscle catabolism.

10.1128/msystems.00682-22.3 FIG S3 Immunodeficient mice exhibited changes in skeletal muscle catabolism metabolites after infection with the *rtxA1* deletion mutant. Mice were treated with or without CPA and treated with PBS or infected with V. vulnificus (WT and Δ*rtxA1*) strains. The samples were divided into six groups as follows: PBS treatment, PBS plus CPA treatment, V. vulnificus infection (Vv), V. vulnificus infection and cyclophosphamide treatment (Vv+CPA), MARTX (Δ*rtxA1*) mutant infection, and MARTX mutant infection and CPA treatment (Δ*rtxA1+*CPA). (A) Hematoxylin and eosin staining showed the effect of PBS treatment or infection with the V. vulnificus (WT, DKO, Δ*rtxA1*, and Δ*vvhA*) strains. (B) The changes in the expression levels of the *MIP-2*, *TNF-α*, and *IL-6* mRNAs triggered by infection with V. vulnificus WT or mutant (Δ*rtxA1,* Δ*vvhA*, and DKO) strains were estimated by RT-qPCR. The expression of each mRNA was normalized to that of the *GAPDH* mRNA (3 to 5/group). (C) The number of bacteria in skeletal muscle tissues was estimated using a CFU assay. These skeletal muscles were infected with V. vulnificus (WT, DKO, Δ*rtxA1*, and Δ*vvhA*) strains for 1, 3, and 9 h. The asterisks denote significant differences versus the wild-type group (3 to 5/group). (D) The expression levels of the 16S rRNA transcript were estimated by RT-qPCR (3 to 5/group). (E) White blood cells were counted in mouse whole blood (*n* = 4/group). (F) The expression levels of the 16S rRNA transcript were estimated by RT-qPCR (4/group). (G) The number of bacteria in whole blood was estimated using a CFU assay. Skeletal muscles were infected for 6 h (4/group). (H) Hematoxylin and eosin staining showed the effects of PBS treatment or infection with V. vulnificus (WT and Δ*rtxA1*) strains on mouse skeletal muscles treated with or without CPA. (I) A Western blot analysis of myeloperoxidase in mouse skeletal muscles was conducted at 6 h of infection. (J) Metabolites of creatine and creatinine in skeletal muscles were extracted from the CE-TOFMS data at 6 h of infection (4/group). *, *P *< 0.05, and **, *P *< 0.01, versus the control group (*t* test). *P* values were determined by one-way ANOVA followed by Tukey’s test. Different letters indicate significant difference. A *P* value of <0.05 is considered significant. Download FIG S3, TIF file, 2.6 MB. Copyright © 2023 Ishida et al. 2023 Ishida et al. https://creativecommons.org/licenses/by/4.0/ This content is distributed under the terms of the Creative Commons Attribution 4.0 International license.

### MARTX does not cause TCA cycle metabolite changes directly.

**FIG 4:**
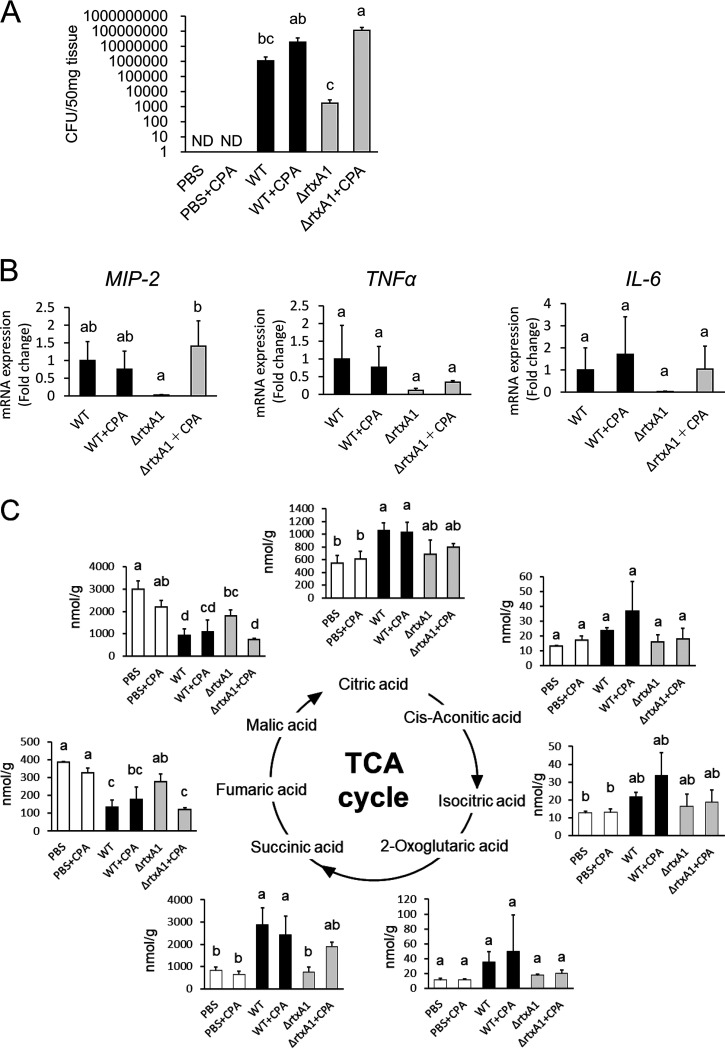
Infection with the *rtxA1* deletion mutant yielded metabolic changes similar to those detected for the wild-type strain in CPA-treated mice. Mice were treated with or without CPA and stimulated with PBS or V. vulnificus (WT and Δ*rtxA1* strains). The mice were divided into six groups, as follows: PBS treatment, PBS with CPA treatment, V. vulnificus infection (Vv), V. vulnificus infection and CPA treatment (Vv+CPA), MARTX (Δ*rtxA1*) mutant infection, and MARTX mutant infection and CPA treatment (Δ*rtxA1+*CPA). (A) The number of bacteria in skeletal muscle tissues was estimated using a CFU assay after 6 h of infection (4/group). (B) Expression levels of *MIP-2*, *TNF-α*, and *IL-6* mRNAs were estimated by reverse transcription-quantitative PCR (RT-qPCR). The expression of each mRNA was normalized to that of the *GAPDH* mRNA (4/group). (C) Concentrations of the TCA cycle metabolites in skeletal muscles were measured by CE-TOFMS 6 h after the treatments (3 or 4/group). Each bar indicates the mean concentration. *P* values were determined by one-way ANOVA followed by Tukey’s test. Different letters indicate significant differences (*P* < 0.05).

According to the data presented in Fig. S3C, the wild-type and *vvhA* deletion mutant strains yielded a gradual increase in bacterial cell counts in mouse skeletal muscle, whereas the *rtxA1* deletion mutant strain did not (Fig. S3C). Similarly, the mRNA levels of the bacterial 16S rRNA were decreased in *rtxA1* deletion mutants in infected skeletal muscle (Fig. S3D). These data indicate that the *rtxA1* deletion mutant strain was removed from the infected site by the host immune system. V. vulnificus may cause metabolic changes in the TCA cycle via two possible mechanisms: changes to the host metabolic system directly by MARTX or the contribution by MARTX to the metabolic changes via the promotion of infection by evading the host immune system. We evaluated these hypotheses using neutropenic mice treated with cyclophosphamide monohydrate (CPA). We examined the number of white blood cells (Fig. S3E) and living bacterial cells (Fig. 4A; Fig. S3F and G) in each group. The number of white blood cells was decreased in the CPA-treated mice, and the bacterial cell count of the Δ*rtxA1* mutant in CPA-treated mice was not decreased at 6 h of infection. Similarly, the mRNA levels of 16S rRNA were elevated in the Δ*rtxA1*+CPA group in infected skeletal muscle. Moreover, living bacteria were detected in mouse blood in the Vv, Vv+CPA, and Δ*rtxA1*+CPA groups. These data indicate that the Δ*rtxA1* mutant was affected by treatment with CPA in skeletal muscle. Next, we assessed inflammation in CPA-treated mice using hematoxylin-and-eosin (H&E) staining (Fig. S3H) and Western blotting (Fig. S3I). Histopathological changes and myeloperoxidase protein levels indicated the migration of neutrophils in normal mice, whereas CPA-treated mice did not exhibit migration of neutrophils. In addition, the reduction in the expression levels of the *MIP-2* mRNA in infection with the Δ*rtxA1* strain was recovered by CPA treatment in skeletal muscle (Fig. 4B). These results suggest that MARTX contributes to evasion of the clearance of the bacteria by the host immune system and that CPA treatment enables the infection of the host skeletal muscle, even for the *rtxA1* mutant.

Under these conditions, we checked the metabolic changes in the creatine-to-creatinine pathway (Fig. S3J) and the TCA cycle (Fig. 4C). The levels of metabolites in skeletal muscle infected with wild-type (WT) and Δ*rtxA1* strains were reduced significantly in the CPA-treated mice. Moreover, the V. vulnificus-mediated TCA metabolic changes were abolished in mice infected with the Δ*rtxA1* strain but were recovered in CPA-treated mice. These data indicate that MARTX does not change the TCA metabolism directly. However, MARTX was necessary for the infiltration, survival, and replication of bacterial cells in skeletal muscle, and bacterial infection caused changes in metabolites in the TCA cycle and catabolism.

### Metabolic changes in mouse skeletal muscle during *Vibrio* infection.

**FIG 5:**
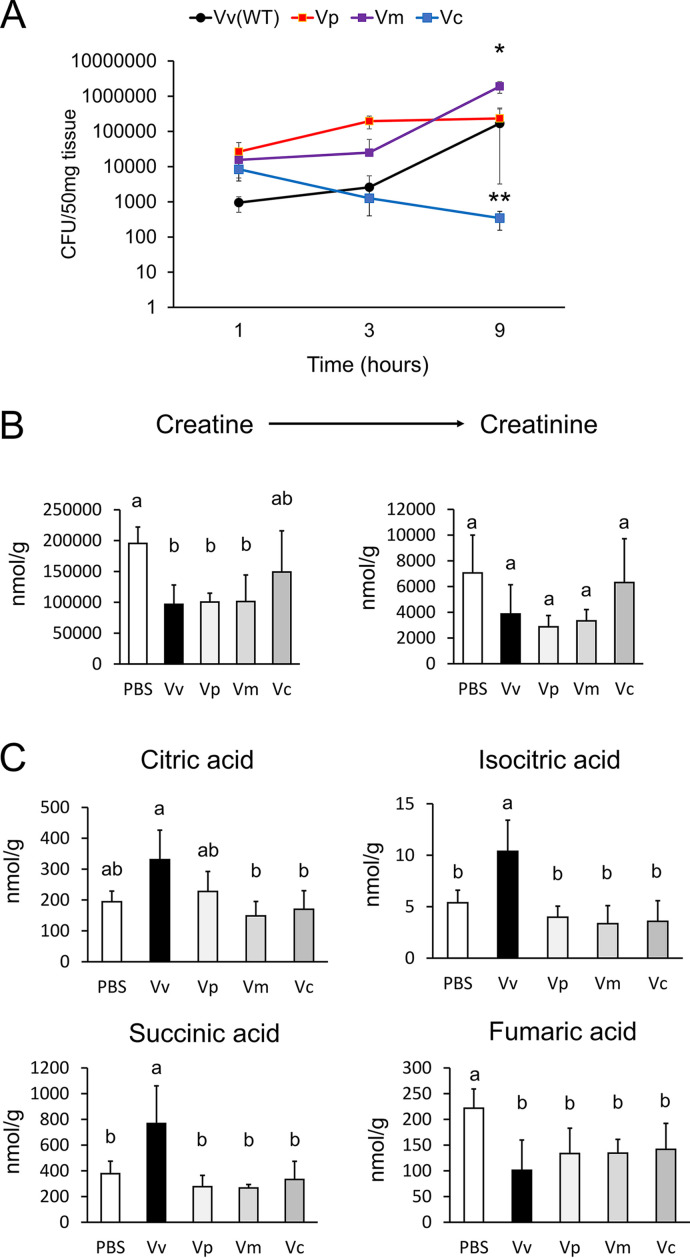
Other *Vibrio* spp. affect the host metabolism in mouse skeletal muscle. (A) The number of bacteria in skeletal muscles was estimated using the CFU assay. These skeletal muscles were infected with V. vulnificus (Vv), Vibrio parahaemolyticus (Vp), Vibrio mimicus (Vm), or Vibrio cholerae (Vc) for 9 h. The asterisks denote significant differences versus the Vv group (4/group). (B) Concentrations of arginine, creatine, and creatinine metabolites in skeletal muscles were extracted from CE-TOFMS data regarding treatment with PBS or infection with V. vulnificus, V. parahaemolyticus, *V. mimicus*, or V. cholerae for 9 h (4/group). (C) Concentrations of the citric acid, isocitric acid, succinic acid, and fumaric acid metabolites in skeletal muscle were extracted from CE-TOFMS data for treatment with PBS, V. vulnificus, V. parahaemolyticus, *V. mimicus*, or V. cholerae for 9 h (4/group). Each bar indicates the mean concentration. *, *P *< 0.05, and **, *P *< 0.01, versus the control group (*t* test). *P* values were determined by one-way ANOVA followed by Tukey’s test. Different letters indicate significant differences (*P* < 0.05).

Finally, we confirmed the specificity of the host metabolic changes during V. vulnificus infection. Mice were infected with other *Vibrio* spp. (i.e., Vibrio parahaemolyticus, Vibrio mimicus, and Vibrio cholerae) for 9 h, and the resulting metabolic changes were analyzed (Fig. 5B and C; Fig. S4C). With the analysis of the metabolites, we confirmed the number of living bacteria and the inflammation of skeletal muscles in those infected with *Vibrio* spp. based on histopathological changes and the mRNA expression levels of *MIP-2*, *TNF-α*, and *IL-6* (Fig. 5A; Fig. S4A and B). Our data indicated that V. parahaemolyticus and *V. mimicus* exhibited higher bacterial loads and induced greater inflammatory responses. In contrast, V. cholerae did not show an increase in live bacteria and caused an inflammatory response in infected skeletal muscle. These data implied that these bacteria, known to cause wound infection as observed in V. vulnificus infection, induced metabolic changes in the TCA cycle with the inflammation in skeletal muscle.

10.1128/msystems.00682-22.4 FIG S4 Other *Vibrio spp.* that can infect wounds caused inflammation in mouse skeletal muscles, similar to that observed for V. vulnificus. Mouse skeletal muscles were treated with PBS or infected with V. vulnificus (Vv), V. parahaemolyticus (Vp), *V. mimicus* (Vm), or V. cholerae (Vc) for 9 h. (A) H&E staining showed the effects of PBS treatment or V. vulnificus, V. parahaemolyticus, *V. mimicus*, or V. cholerae infection on mouse skeletal muscles. (B) The expression levels of the *MIP-2*, *TNF-α*, and *IL-6* mRNAs were estimated by RT-qPCR. The expression of each mRNA was normalized to that of the *GAPDH* mRNA (4/group). (C) Metabolites of creatine and creatinine in blood were extracted from the CE-TOFMS data after treatment with PBS or infection with the *Vibrio* spp. for 9 h (4/group). *, *P *< 0.05, and **, *P *< 0.01, versus the control group (*t* test). *P* values were determined by one-way ANOVA followed by Tukey’s test. Different letters indicate significant differences. A *P* value of <0.05 is considered significant. Download FIG S4, TIF file, 8.2 MB. Copyright © 2023 Ishida et al. 2023 Ishida et al. https://creativecommons.org/licenses/by/4.0/ This content is distributed under the terms of the Creative Commons Attribution 4.0 International license.

Next, the mouse skeletal muscle was isolated, and its metabolites were analyzed by CE-TOFMS. The metabolites associated with muscle catabolites, the creatine-to-creatinine pathway, were changed in V. parahaemolyticus- and *V. mimicus*-infected muscle, similar to V. vulnificus infection. Despite the similar catabolic pattern, citric acid, isocitric acid, and succinic acid were not changed in V. parahaemolyticus and *V. mimicus* infection. These results indicated that the increase in TCA cycle metabolites was specific to V. vulnificus-infected skeletal muscle.

## DISCUSSION

Metabolome analyses are instrumental to elucidate host responses to bacterial infection (23, 28). In a previous study, a mouse skeletal muscle infection model was established and revealed a characteristic inflammation after V. vulnificus infection (25, 29). It is known that skeletal muscle has an exacerbated catabolism under conditions of inflammation (30); however, the metabolic changes in the host skeletal muscle in V. vulnificus-infected mice have not been determined. Therefore, we attempted to clarify the metabolic changes in V. vulnificus-infected muscle tissues using CE-TOFMS. Our experimental model also indicated the migration of neutrophils and upregulation of inflammatory cytokines during V. vulnificus infection (Fig. S1A and B). Our metabolome analysis revealed characteristic changes in the TCA cycle and the arginine-to-creatine metabolic pathway after V. vulnificus infection (Fig. 1E). In addition, we found that a V. vulnificus toxin, multifunctional-autoprocessing repeat-in-toxin (MARTX), affected the changes in metabolism simultaneously with the increase in the bacterial counts in skeletal muscle (Fig. 3A and B; Fig. S3C). Importantly, despite the similar creatine and creatinine metabolite levels, the citric acid, isocitric acid, and succinic acid metabolites were not changed in other wound infection models, such as those of V. parahaemolyticus or *V. mimicus* infection (Fig. 5B and C). These results indicated that metabolic changes in the energy metabolic pathway occur in V. vulnificus-infected skeletal muscle.

In general, creatine or creatine-phosphokinase is used as a marker of body muscle injury in clinical blood tests. A previous study reported that the serum creatine-phosphokinase level was increased during V. vulnificus infection (26). In agreement with that study, our data indicated an increase in creatine metabolites in the blood of V. vulnificus-infected mice (Fig. S1E). Conversely, creatine and creatinine metabolites were clearly decreased in V. vulnificus-infected skeletal muscle (Fig. 1A). In turn, skeletal muscles infected with V. parahaemolyticus or *V. mimicus* showed a reduction of creatine and creatinine metabolites, similar to that observed for V. vulnificus infection (Fig. 5B). Moreover, the bacterial counts of V. vulnificus, V. parahaemolyticus, and *V. mimicus* were increased in infected skeletal muscles (Fig. 5A). Taken together, our data suggest that wound infection with *Vibrio* spp. causes the degradation of muscle tissue simultaneously with the propagation of *Vibrio* cells in host tissues and that catabolic metabolites seem to leak into the blood from skeletal muscle. In addition, previous studies reported that the blood creatine phosphokinase level was increased in necrotizing fasciitis by group A beta-hemolytic streptococci (31, 32). Therefore, the measurement of metabolites associated with muscle catabolism is expected to be useful for estimating the seriousness of wound infections, including those caused by *Vibrio* spp., in blood clinical biochemistry tests.

To elucidate the wound infection caused by V. vulnificus, it is necessary to identify the specific metabolic changes triggered by infection with this bacterium. Pathway analysis revealed characteristic changes in metabolites in the TCA cycle (Fig. 1E). In particular, V. vulnificus caused an increase in succinic acid content in muscle tissue, and the metabolic pattern of succinic acid was completely opposite to that of fumaric acid in the TCA cycle (Fig. 2A). In addition, in contrast with that observed for catabolic metabolites, the levels of the citric acid, isocitric acid, and succinic acid metabolites were increased in V. vulnificus-infected skeletal muscle exclusively (Fig. 5C). It is possible that V. vulnificus affects the host energy metabolic pathway to utilize host succinate for survival in host muscle tissues. Recent studies reported that host succinate was used for increasing the virulence of a variety of bacteria. It is known that host succinate activates Salmonella enterica serovar Typhimurium virulence factors. Furthermore, gut microbiota-produced succinate is utilized by a variety of bacteria to enhance their pathogenesis (33, 34). Periodontal ligament cells (PDLSCs) cause succinate accumulation and promote the inflammation triggered by Porphyromonas gingivalis infection (35). Thus, we thought that the catabolism of mouse skeletal muscle triggered by toxins could accelerate the supply of succinate to V. vulnificus, thus enhancing bacterial growth, survival, and virulence activation in host tissues. Moreover, we observed that the expression of the host succinate dehydrogenase mRNA in mitochondria was decreased by V. vulnificus infection (data not shown). These data supported the contention that V. vulnificus promotes succinate accumulation via the downregulation of host succinate dehydrogenase. Taken together, these findings suggest that the inhibition of the succinate-fumarate metabolic pathway provides succinate more effectively to V. vulnificus, with consequent development of dramatic inflammation in infected skeletal muscles.

VVH and MARTX have been correlated with cytotoxicity during host infection (36–39). Therefore, we predicted that VVH and MARTX contribute to the changes in creatine and creatinine metabolites in infected skeletal muscles. Our data indicate that the MARTX deletion mutant alone abolished the changes in the metabolites of muscle catabolism (Fig. S1C; Fig. 3A). Interestingly, TCA cycle metabolites were also abolished in the MARTX deletion mutant simultaneously with the catabolic changes (Fig. 3B). Conversely, V. parahaemolyticus and *V. mimicus* also secrete pore-forming toxins (such as thermostable direct hemolysin [TDH]), which induce permeability, cytotoxicity, and apoptosis in host cells (40–43). According to our data, V. parahaemolyticus and *V. mimicus* caused creatine and creatinine metabolic changes that were similar to those afforded by V. vulnificus (Fig. 5B); however, these pathogens did not affect the TCA cycle (Fig. 5C). These results suggest that the accumulation of energy metabolites in skeletal muscle is associated with MARTX of V. vulnificus, independent of catabolism. A previous study reported that MARTX induces mitochondrion-mediated apoptosis *in vitro* (44). MARTX may cause energy metabolic changes directly, similarly to catabolites. Remarkably, the bacterial counts of the *rtxA1* mutant strain in skeletal muscle gradually decreased over time in our infection model (Fig. S3C and D). A previous study reported that MARTX impaired the ability of host phagocytes to clear V. vulnificus infection (45). We considered that MARTX had a role in evasion of the host clearance system. Therefore, to investigate the exact contribution of bacterial proliferation in the metabolic change of infected skeletal muscle, we used a neutropenic mouse model of infection with a MARTX deletion mutant. During infection with the *rtxA1* mutant strain in the neutropenic mouse model, the creatine and creatinine metabolites and the expression levels of inflammatory cytokines were similar to those observed for infection with the WT strain, and the bacterial cell counts of the *rtxA1* strain were recovered in the neutropenic mice (Fig. 4A and B; Fig. S3E to J). Based on these results, we concluded that MARTX contributes to host metabolic changes by suppressing the host immune system and promoting proliferation in infected skeletal muscles.

In summary, we identified characteristic metabolic changes in protein catabolism and the TCA cycle triggered by V. vulnificus infection. MARTX contributed to metabolic changes by suppressing the host immune system and promoting bacterial cell infiltration into the host skeletal muscle during wound infection. Based on our results, we propose that the metabolic changes in infected skeletal muscles were correlated with dramatic inflammatory response triggered by V. vulnificus infection. However, the virulence factors that directly affect host metabolism have not been clarified; therefore, additional research is needed to identify the virulence factors associated with these metabolic changes. Future studies focusing on metabolic changes can be used to assist in the design of new therapeutic strategies and elucidate the mechanism of V. vulnificus wound infection and the resulting characteristic inflammation.

## MATERIALS AND METHODS

### Bacterial strains and culture conditions.

Vibrio vulnificus strains (wild-type, VVH [Δ*vvhA*] deletion mutant, MARTX deletion [Δ*rtxA1*] mutant, and DKO strains) derived from CMCP6 were provided by Takashige Kashimoto at Kitasato University (46, 47), and the V. parahaemolyticus, V. cholerae, and *V. mimicus* strains were obtained from the Research Institute for Microbial Diseases (RIMD), Osaka University. The bacterial strains used in this experiment are listed in Table 1. V. vulnificus strains were cultured in 2% NaCl–LB broth (1% tryptone, 0.5% yeast extract), V. parahaemolyticus was cultured in 3% NaCl–LB broth, and *V. mimicus* and V. cholerae were cultured in 1% NaCl–LB broth overnight at 37°C with shaking (170 rpm). Bacterial culture media were centrifuged at 12,000 rpm for 2 min and concentrated, and organisms were grown on thiosulfate-citrate-bile salts-sucrose (TCBS) agar overnight. A single colony was picked from the TCBS agar and cultured in LB broth for 8 h. The bacterial suspension was mixed with a glycerol solution (final concentration of glycerol, 15%) and stored at −80°C. For experiments, bacterial strains were picked from the stock and were cultured in 1%, 2%, or 3% LB broth overnight. Subsequently, the culture broth was recultured in new LB broth for 2 h and then concentrated to an optical density at 600 nm (OD_600_) of 1.0 with PBS.

**TABLE 1:** *Vibrio* species used in this study

Species	Strain or genotype	Description	Source (references)
Vibrio vulnificus	CMCP6	V. vulnificus wild type, clinical isolate	Kitasato University (46, 47)
	*ΔvvhA*, *rtxA1*(DKO)	VV2_0404 and VV2_0479 deletion mutant derived from CMCP6	Kitasato University (46, 47)
	Δ*rtxA1*	VV2_0479 deletion mutant derived from CMCP6	Kitasato University (46, 47)
	Δ*vvhA*	VV2_0404 deletion mutant derived from CMCP6	Kitasato University (46, 47)
Vibrio parahaemolyticus	RIMD2210633	V. parahaemolyticus wild type, clinical isolate	RIMD, Osaka University
Vibrio mimicus	RIMD2218070	*V. mimicus* wild type, clinical isolate	RIMD, Osaka University
Vibrio cholerae	RIMD2203102	V. cholerae wild type, clinical isolate	RIMD, Osaka University

### Animals and infection.

We purchased 5-week-old male Crl:CD1 mice (Charles River Laboratories Japan, Kanagawa, Japan). The mice were housed in a controlled environment with a 12-h/12-h light/dark cycle and maintained at 23°C. One week later, the mice were utilized in the animal experiments.

Previously, Yamazaki et al. established a V. vulnificus mouse wound infection model (25). Mice were subcutaneously inoculated with 100 μL of a 10×-diluted bacterial solution adjusted to an OD_600_ of 1.0 into the right caudal thighs and infected (10^6^ CFU/head) at 23°C after anesthesia using isoflurane. The mice were sacrificed 9 h after the injections. The mouse muscles were dissected and placed in a 1.5-mL tube. Mouse blood samples were collected in 1.5-mL tubes and EDTA dipotassium (EDTA-2K) at 1 mg/mL was added, to prevent blood coagulation.

Neutropenic mice, 6 weeks old, were prepared via a 2-dose intraperitoneal administration of cyclophosphamide monohydrate at 150 mg/kg of body weight at day −4 and 100 mg/kg at day −1 from infection. Neutropenic mice were infected with bacteria using the method used in another experiment. The mice were sacrificed 6 h after the injections. The mouse muscles were dissected and placed in 1.5-mL tubes. Mouse blood samples were collected in 1.5-mL tubes, and EDTA-2K at 1 mg/mL was added to prevent blood coagulation.

### Metabolome analysis of skeletal muscles and blood by capillary electrophoresis time-of-flight mass spectrometry.

The tissues were frozen rapidly in liquid nitrogen and stored at −80°C. The skeletal muscles were cut on ice, and 50-mg samples were collected in 1.5-mL tubes. The skeletal muscles were mixed with 500 μL of methanol containing 10 μM methionine sulfone and camphor-10-sulfonic acid, as an internal control, in each tube. The muscles were homogenized using a beads cell disrupter (Micro Smash, MS-100R; Tomy) and the lysates were removed into a new tube. The blood (50 μL) was placed in 2-mL tubes and mixed with 500 μL of methanol containing 10 μM methionine sulfone and camphor-10-sulfonic acid, as an internal control, in each tube. These samples were mixed with 200 μL of ultrapure water and 500 μL of chloroform. After vortex mixing, the samples were centrifuged at 9,100 × *g* for 5 min at 4°C, and the supernatant was filtered using ultracentrifugal 5-kDa-cutoff filters (Millipore) at 9,100 × *g* for 4 h at 4°C. The filtrates were concentrated in a vacuum evaporator and dissolved with ultrapure water containing internal controls (3-aminopyrrolidine, *N*,*N*-diethyl-2-phenylacetamide, trimesic acid, and 3-hydroxynaphthalene-2,7-disulfonic acid).

The samples were analyzed using Agilent 7100 capillary electrophoresis (CE) system coupled with an Agilent 6230 TOF LC-MS (Agilent Technologies, Palo Alto, CA) by Human Metabolome Technologies, Inc. (HMT, Tsuruoka, Japan). CE-TOFMS analysis of cationic and anionic metabolites was performed as described previously (48–51). Cationic metabolites were analyzed with a fused silica capillary (50 μm [internal diameter] by 80 cm [total length]) with cationic electrophoresis buffer (H3302-2031; HMT) as the electrolyte. The sample solution was injected at a pressure of 5,000 Pa for 10 s. The applied voltage was set at 30 kV. Anionic metabolites were analyzed with a fused silica capillary (50 μm by 80 cm) with anionic electrophoresis buffer (H3301-2031, HMT) as the electrolyte. The sample solution was injected at a pressure of 5,000 Pa for 10 s. The applied voltage was set at −30 kV. TOF LC-MS was conducted in the positive-ion mode (4,000 V) and the negative-ion mode (4,000 V) for cationic and anionic metabolites, respectively. Exact mass data were scanned at a rate of 1.5 cycles/s over a 50-to-1,000 *m/z* range.

Analyzed raw data were processed using Agilent MassHunter qualitative analysis (version B.07.00). Each metabolite was identified and quantified based on the reference peak information, including *m/z*, migration time, and peak area to compare with the standard (Human Metabolome Technologies, Inc., Tsuruoka, Japan; HMT-H3304-3031). Each peak area was normalized based on internal standard levels, and then the resultant relative area values were normalized by sample amounts to obtain a relative level for each metabolite. Methionine sulfone was used for the density correction of cationic metabolites, and camphor-10-sulfonic acid was used for the density correction of anionic metabolites.

### Detection of living *Vibrio* cells in mouse skeletal muscles and blood.

The homogenized Mouse skeletal muscles were weighed to 100 mg, added to 900 μL of PBS, homogenized using a homogenizer pestle, and diluted gradually (10^−1^ to 10^−5^). Mouse blood was collected in 100-μL portions in 1.5-mL tubes and EDTA-2K at 1 mg/mL was added to prevent blood coagulation. These skeletal muscles and blood samples with infection with V. vulnificus strains were plated at 100 μL on 2% LB agar containing 50 μg/mL rifampicin, followed by incubation for 12 h at 37°C. V. parahaemolyticus was plated at 100 μL on 3% LB agar and incubated for 12 h at 37°C. *V. mimicus* and V. cholerae were plated at 100 μL on 1% LB agar and incubated for 12 h at 37°C. Live bacterial cells were counted by calculating the number of CFU/50 mg of skeletal muscle or CFU/100 μL of blood.

### Quantitative real-time PCR analysis.

Total RNA was isolated from tissue homogenates using the TRIzol reagent (Thermo Fisher Scientific Inc, Massachusetts, USA). cDNA was synthesized using the Primescript RT reagent kit (TaKaRa, Kyoto, Japan). Gene expression levels were measured using quantitative real-time PCR with TB Green premix Ex Taq (TaKaRa, Kyoto, Japan). The mouse-specific primer pairs used in this experiment are listed in Table 2 (52, 53).

**TABLE 2:** Primers used in this study to estimate expression levels of inflammation-associated genes

Gene	Sequence (5′–3′)	Reference
*MIP-2*	F: GCCAAGGGTTGACTTCAAGA	48
	R: CTTCAGGGTCAAGGCAAACT	48
*IL-6*	F: GCCAGAGTCCTTCAGAGAGA	This study
	R: TGGTCCTTAGCCACTCCTTC	This study
*TNF-α*	F: GTGCCTATGTCTCAGCCTCT	This study
	R: CTGATGAGAGGGAGGCCATT	This study
*16S*	F: GTTGTGAGGAAGGTGGTGTC	This study
	R: CCGGGCTTTCACATCTGAC	This study
*GAPDH*	F: GGTGGTCTCCTCTGACTTCAACA	49
	R: GTGGTCGTTGAGGGCAATG	49

### H&E staining.

Muscle tissues were fixed in 4% paraformaldehyde for 24 h, washed in PBS, and embedded in paraffin. Two-micrometer sections were stained with H&E according to standard procedures.

### Measurement of white blood cell counts.

Mouse whole blood was collected in 10-μL portions in 1.5-mL tubes, and the number of white blood cells was measured using an automatic blood cell counter for animals (Microsemi LC-662; Horiba Medical, Kyoto, Japan).

### Western blotting.

Mouse skeletal muscles were added to 500 μL of radio immunoprecipitation assay (RIPA) buffer (pH 7.4; 50 mM Tris HCl, 150 mM NaCl, 1 mM EDTA, 1% sodium deoxycholate, 0.1% SDS, 1% Triton X-100) per 10 mg of muscle tissue and homogenized sufficiently using a homogenizer (4,500 rpm, 4°C, 1 min). The mixture was centrifuged (12,000 rpm, 4°C, 20 min) and the supernatant was collected. The supernatant was then mixed with 5× sample buffer (pH 6.8; 250 mM Tris-HCl, 50% glycerol, 5% SDS, bromophenol blue, 25% 2-mercaptoethanol) and boiled for 5 min at 95°C. Equal amounts of protein were loaded on SDS polyacrylamide gels, separated by electrophoresis, and transferred to polyvinylidene difluoride membranes, which were then blocked with Tris-buffered saline containing Tween 20 (TBS-T; pH 7.6; 20 mM Tris, 150 mM NaCl, 0.02% polyoxymethylene 20 sorbitan monolaurate) for 1 h at 23°C and incubated with the primary antibodies overnight at 4°C. Subsequently, the membranes were washed 3 times with TBS-T, incubated with immunoglobulin G (IgG)–horseradish peroxidase-conjugated secondary antibodies for 2 h at 23°C, washed 3 times with TBS-T, visualized using the enhanced chemiluminescence (ECL) Western blotting kit (GE Healthcare Bio-Sciences Corp., New Jersey, USA), and imaged using LASS-2000. Antibodies against myeloperoxidase (MPO) and glyceraldehyde-3-phosphate dehydrogenase (GAPDH) were purchased from Santa Cruz Biotechnology, Inc. (Texas, USA). Horseradish peroxidase-conjugated goat anti-rabbit IgG was from Medical & Biological Laboratories Co., Ltd. (Nagoya, Japan).

### Statistical analysis.

All studies were conducted in triplicate and on 3 separate days. The data are presented as means and standard errors of the means (SEM) for all experiments. *P* values were calculated using the data presented as the means and SEM for all experiments. Hierarchical clustering by heat map and PCA of metabolite data sets were computed using MassHunter Mass Profiler Professional (Agilent Technologies, Palo Alto, CA). *P* values were calculated using Student's *t* test with the threshold for significance set at a *P* value of <0.05. For metabolic analysis, comparisons between groups were estimated by Dunnett’s test with Benjamini-Hochberg normalization using IBM SPSS Statistics 25 (SPSS Inc., Chicago, IL, USA). A Benjamini-Hochberg false discovery rate (FDR; *q* value) of <0.05 was considered statistically significant. Multiple data analysis was performed based on one-way analysis of variance (ANOVA) and Tukey’s honestly significant difference test using IBM SPSS Statistics 25 (SPSS Inc., Chicago, IL, USA). A *P* value of <0.05 was considered significant.

### Data availability.

The data from metabolic analysis using CE-MS are publicly available at EMBL-EBI’s MetaboLights repository (https://www.ebi.ac.uk/metabolights) with the data set identifier MTBLS6837; these data are summarized in Table S1.

## References

[1] Dechet AM, Yu PA, Koram N, Painter J. 2008. Nonfoodborne Vibrio infections: an important cause of morbidity and mortality in the United States, 1997–2006. Clin Infect Dis 46:970–976. doi:10.1086/529148. 18444811

[2] Heng SP, Letchumanan V, Deng CY, Ab Mutalib NS, Khan TM, Chuah LH, Chan KG, Goh BH, Pusparajah P, Lee LH. 2017. Vibrio vulnificus: an environmental and clinical burden. Front Microbiol 8:997. doi:10.3389/fmicb.2017.00997. 28620366 PMC5449762

[3] Oliver JD. 2015. The biology of Vibrio vulnificus. Microbiol Spectr 3:VE-0001-2014. doi:10.1128/microbiolspec.VE-0001-2014. 26185084

[4] Baker-Austin C, Oliver JD. 2018. Vibrio vulnificus : new insights into a deadly opportunistic pathogen. Environ Microbiol 20:423–430. doi:10.1111/1462-2920.13955. 29027375

[5] Leng F, Lin S, Wu W, Zhang J, Song J, Zhong M. 2019. Epidemiology, pathogenetic mechanism, clinical characteristics, and treatment of Vibrio vulnificus infection: a case report and literature review. Eur J Clin Microbiol Infect Dis 38:1999–2004. doi:10.1007/s10096-019-03629-5. 31325061

[6] Nazir S, Brown K, Shin AK, Donato AA. 2016. Vibrio vulnificus infection and liver cirrhosis: a potentially lethal combination. BMJ Case Rep 2016:bcr2016214772. doi:10.1136/bcr-2016-214772. PMC4885366 27151052

[7] Ramos LB, Darwin L-C, de-Leon AP. 2021. A fatal case of Vibrio vulnificus septicemia in an end-stage liver disease patient. Enferm Infecc Microbiol Clin (Engl Ed) 39:352–354. doi:10.1016/j.eimce.2020.11.019. 34353513

[8] Jones MK, Oliver JD. 2009. Vibrio vulnificus: disease and pathogenesis. Infect Immun 77:1723–1733. doi:10.1128/IAI.01046-08. 19255188 PMC2681776

[9] Horseman MA, Surani S. 2011. A comprehensive review of Vibrio vulnificus: an important cause of severe sepsis and skin and soft-tissue infection. Int J Infect Dis 15:e157–e166. doi:10.1016/j.ijid.2010.11.003. 21177133

[10] Hernández-Cabanyero C, Amaro C. 2020. Phylogeny and life cycle of the zoonotic pathogen Vibrio vulnificus. Environ Microbiol 22:4133–4148. doi:10.1111/1462-2920.15137. 32567215

[11] Huang KC, Weng HH, Yang TY, Chang TS, Huang TW, Lee MS. 2016. Distribution of fatal Vibrio vulnificus necrotizing skin and soft-tissue infections a systematic review and meta-analysis. Medicine (Baltimore) 95:e2627. doi:10.1097/MD.0000000000002627. 26844475 PMC4748892

[12] Jeong HG, Satchell KJF. 2012. Additive function of vibrio vulnificus MARTXVv and VvhA cytolysins promotes rapid growth and epithelial tissue necrosis during intestinal infection. PLoS Pathog 8:e1002581. doi:10.1371/journal.ppat.1002581. 22457618 PMC3310748

[13] Lee SJ, Lee HJ, Jung YH, Kim JS, Choi SH, Han HJ. 2018. Melatonin inhibits apoptotic cell death induced by Vibrio vulnificus VvhA via melatonin receptor 2 coupling with NCF-1 article. Cell Death Dis 9:48. doi:10.1038/s41419-017-0083-7. 29352110 PMC5833450

[14] Song EJ, Lee SJ, Lim HS, Kim JS, Jang KK, Choi SH, Han HJ. 2016. Vibrio vulnificus VvhA induces autophagy-related cell death through the lipid raft-dependent c-Src/NOX signaling pathway. Sci Rep 6:27080. doi:10.1038/srep27080. 27250250 PMC4890043

[15] Kim BS. 2018. The modes of action of MARTX toxin effector domains. Toxins (Basel) 10:507. doi:10.3390/toxins10120507. 30513802 PMC6315884

[16] Gavin HE, Beubier NT, Satchell KJF. 2017. The effector domain region of the Vibrio vulnificus MARTX toxin confers biphasic epithelial barrier disruption and is essential for systemic spread from the intestine. PLoS Pathog 13:e1006119. doi:10.1371/journal.ppat.1006119. 28060924 PMC5218395

[17] Toma C, Higa N, Koizumi Y, Nakasone N, Ogura Y, McCoy AJ, Franchi L, Uematsu S, Sagara J, Taniguchi S, Tsutsui H, Akira S, Tschopp J, Núñez G, Suzuki T. 2010. Pathogenic Vibrio activate NLRP3 inflammasome via cytotoxins and TLR/nucleotide-binding oligomerization comain-mediated NF-κB signaling. J Immunol 184:5287–5297. doi:10.4049/jimmunol.0903536. 20348425

[18] Lee C-T, Pajuelo D, Llorens A, Chen Y-H, Leiro JM, Padrós F, Hor L-I, Amaro C. 2013. MARTX of Vibrio vulnificus biotype 2 is a virulence and survival factor. Environ Microbiol 15:419–432. doi:10.1111/j.1462-2920.2012.02854.x. 22943291

[19] Lo H-R, Lin J-H, Chen Y-H, Chen C-L, Shao C-P, Lai Y-C, Hor L-I. 2011. RTX toxin enhances the survival of Vibrio vulnificus during infection by protecting the organism from phagocytosis. J Infect Dis 203:1866–1874. doi:10.1093/infdis/jir070. 21422475

[20] Lee BC, Choi SH, Kim TS. 2008. Vibrio vulnificus RTX toxin plays an important role in the apoptotic death of human intestinal epithelial cells exposed to Vibrio vulnificus. Microbes Infect 10:1504–1513. doi:10.1016/j.micinf.2008.09.006. 18849006

[21] Kim YR, Lee SE, Kang IC, Nam KIL, Choy HE, Rhee JH. 2013. A bacterial RTX toxin causes programmed necrotic cell death through calcium-mediated mitochondrial dysfunction. J Infect Dis 207:1406–1415. doi:10.1093/infdis/jis746. 23225896

[22] Kim BS, Kim J-H, Choi S, Park S, Lee E-Y, Koh S, Ryu C-M, Kim S-Y, Kim MH. 2020. MARTX toxin-stimulated interplay between human cells and Vibrio vulnificus. mSphere 5:e00659-20. doi:10.1128/mSphere.00659-20. 32817457 PMC7426173

[23] Afzal M, Saccenti E, Madsen MB, Hansen MB, Hyldegaard O, Skrede S, Martins dos Santos VAP, Norrby-Teglund A, Svensson M. 2020. Integrated univariate, multivariate, and correlation-based network analyses reveal metabolite-specific effects on bacterial growth and biofilm formation in necrotizing soft tissue infections. J Proteome Res 19:688–698. . doi:10.1021/acs.jproteome.9b00565. 31833369

[24] Ledwaba SE, Costa DVS, Bolick DT, Giallourou N, Medeiros PHQS, Swann JR, Traore AN, Potgieter N, Nataro JP, Guerrant RL. 2020. Enteropathogenic Escherichia coli infection induces diarrhea, intestinal damage, metabolic alterations, and increased intestinal permeability in a murine model. Front Cell Infect Microbiol 10:595266. doi:10.3389/fcimb.2020.595266. 33392105 PMC7773950

[25] Yamazaki K, Kashimoto T, Morita M, Kado T, Matsuda K, Yamasaki M, Ueno S. 2019. Identification of in vivo essential genes of Vibrio vulnificus for establishment of wound infection by signature-tagged mutagenesis. Front Microbiol 10:123. doi:10.3389/fmicb.2019.00123. 30774628 PMC6367243

[26] Nakafusa J, Misago N, Miura Y, Kayaba M, Tanaka T, Narisawa Y. 2001. The importance of serum creatine phosphokinase level in the early diagnosis, and as a prognostic factor, of vibrio vulnificus infection. Br J Dermatol 145:280–284. doi:10.1046/j.1365-2133.2001.04347.x. 11531792

[27] Ilaiwy A, Ten Have GAM, Bain JR, Muehlbauer MJ, O'Neal SK, Berthiaume JM, Parry TL, Deutz NE, Willis MS. 2019. Identification of metabolic changes in ileum, jejunum, skeletal muscle, liver, and lung in a continuous i.v. Pseudomonas aeruginosa model of sepsis using nontargeted metabolomics analysis. Am J Pathol 189:1797–1813. doi:10.1016/j.ajpath.2019.05.021. 31439155 PMC6723233

[28] Nurdalila AA, Mayalvanan Y, Baharum SN. 2019. Metabolite profiling of Epinephelus fuscoguttatus infected with vibriosis reveals Omega 9 as potential metabolite biomarker. Fish Physiol Biochem 45:1203–1215. doi:10.1007/s10695-019-00633-6. 30915615

[29] Qin K, Fu K, Liu J, Wu C, Wang Y, Zhou L. 2019. Vibrio vulnificus cytolysin induces inflammatory responses in RAW264.7 macrophages through calcium signaling and causes inflammation in vivo. Microb Pathog 137:103789. doi:10.1016/j.micpath.2019.103789. 31605759

[30] Ono Y, Saito M, Fujinami Y, Inoue S, Kotani J. 2020. Skeletal muscle protein catabolism, protein anabolism, and myogenesis after various types of insults. Surg Metab Nutr 54:139–142. doi:10.11638/jssmn.54.3_139.

[31] Yoshizawa S, Matsumura T, Ikebe T, Ichibayashi R, Fukui Y, Satoh T, Tsubota T, Honda M, Ishii Y, Tateda K, Ato M. 2019. Streptococcal toxic shock syndrome caused by β-hemolytic streptococci: clinical features and cytokine and chemokine analyses of 15 cases. J Infect Chemother 25:355–361. doi:10.1016/j.jiac.2019.01.006. 30744988

[32] Simonart T, Nakafusa J, Narisawa Y. 2004. The importance of serum creatine phosphokinase level in the early diagnosis and microbiological evaluation of necrotizing fasciitis. J Eur Acad Dermatol Venereol 18:687–690. doi:10.1111/j.1468-3083.2004.01108.x. 15482296

[33] Ferreyra JA, Wu KJ, Hryckowian AJ, Bouley DM, Weimer BC, Sonnenburg JL. 2014. Gut microbiota-produced succinate promotes C. difficile infection after antibiotic treatment or motility disturbance. Cell Host Microbe 16:770–777. doi:10.1016/j.chom.2014.11.003. 25498344 PMC4859344

[34] Curtis MM, Hu Z, Klimko C, Narayanan S, Deberardinis R, Sperandio V. 2014. The gut commensal bacteroides thetaiotaomicron exacerbates enteric infection through modification of the metabolic landscape. Cell Host Microbe 16:759–769. doi:10.1016/j.chom.2014.11.005. 25498343 PMC4269104

[35] Su W, Shi J, Zhao Y, Yan F, Lei L, Li H. 2020. Porphyromonas gingivalis triggers inflammatory responses in periodontal ligament cells by succinate-succinate dehydrogenase–HIF-1α axis. Biochem Biophys Res Commun 522:184–190. doi:10.1016/j.bbrc.2019.11.074. 31757417

[36] Kashimoto T, Akita T, Kado T, Yamazaki K, Ueno S. 2017. Both polarity and aromatic ring in the side chain of tryptophan 246 are involved in binding activity of Vibrio vulnificus hemolysin to target cells. Microb Pathog 109:71–77. doi:10.1016/j.micpath.2017.05.029. 28546115

[37] Sugiyama H, Kashimoto T, Ueno S, Susa N. 2013. Inhibition of binding of Vibrio vulnificus hemolysin (VVH) by MβCD. J Vet Med Sci 75:649–652. doi:10.1292/jvms.12-0387. 23238452

[38] Antic I, Biancucci M, Satchell KJF. 2014. Cytotoxicity of the Vibrio vulnificus MARTX toxin effector DUF5 is linked to the C2A subdomain. Proteins Struct Funct Bioinforma 82:2643–2656. doi:10.1002/prot.24628. PMC4177264 24935440

[39] Guo RH, Im YJ, Shin SI, Jeong K, Rhee JH, Kim YR. 2019. Vibrio vulnificus RtxA1 cytotoxin targets filamin A to regulate PAK1- and MAPK-dependent cytoskeleton reorganization and cell death. Emerg Microbes Infect 8:934–945. doi:10.1080/22221751.2019.1632153. 31237474 PMC6598492

[40] Makino K, Oshima K, Kurokawa K, Yokoyama K, Uda T, Tagomori K, Iijima Y, Najima M, Nakano M, Yamashita A, Kubota Y, Kimura S, Yasunaga T, Honda T, Shinagawa H, Hattori M, Iida T. 2003. Genome sequence of Vibrio parahaemolyticus: a pathogenic mechanism distinct from that of V cholerae. Lancet 361:743–749. doi:10.1016/S0140-6736(03)12659-1. 12620739

[41] Naim R, Yanagihara I, Iida T, Honda T. 2001. Vibrio parahaemolyticus thermostable direct hemolysin can induce an apoptotic cell death in Rat-1 cells from inside and ouside of the cells. FEMS Microbiol Lett 195:237–244. doi:10.1111/j.1574-6968.2001.tb10527.x. 11179658

[42] Yoshida H, Honda T, Miwatani T. 1991. Purification and characterization of a hemolysin of Vibrio mimicus that relates to the thermostable direct hemolysin of Vibrio parahaemolyticus. FEMS Microbiol Lett 84:249–254. doi:10.1111/j.1574-6968.1991.tb04605.x. 1804756

[43] Nishibuchi M, Khaemonee-Iam V, Honda T, Kaper JB, Miwatani T. 1990. Comparative analysis of the hemolysin genes of Vibrio cholerae non-O1, V. mimicus, and V. hollisae that are similar to the tdh gene of V. parahaemolyticus. FEMS Microbiol Lett 67:251–256. doi:10.1111/j.1574-6968.1990.tb04028.x. 2323548

[44] Agarwal S, Zhu Y, Gius DR, Satchell KJF. 2015. The Makes Caterpillars Floppy (MCF)-like domain of Vibrio vulnificus induces mitochondrion-mediated apoptosis. Infect Immun 83:4392–4403. doi:10.1128/IAI.00570-15. 26351282 PMC4598404

[45] Chen CL, Chien SC, Leu TH, Harn HIC, Tang MJ, Hor LI. 2017. Vibrio vulnificus MARTX cytotoxin causes inactivation of phagocytosis-related signaling molecules in macrophages. J Biomed Sci 24:58. doi:10.1186/s12929-017-0368-2. 28822352 PMC5563386

[46] Kashimoto T, Ueno S, Koga T, Fukudome S, Ehara H, Komai M, Sugiyama H, Susa N. 2010. The aromatic ring of phenylalanine 334 is essential for oligomerization of Vibrio vulnificus hemolysin. J Bacteriol 192:568–574. doi:10.1128/JB.01049-09. 19897654 PMC2805311

[47] Yamazaki K, Kashimoto T, Kado T, Yoshioka K, Ueno S. 2022. Increased vascular permeability due to spread and invasion of Vibrio vulnificus in the wound infection exacerbates potentially fatal necrotizing disease. Front Microbiol 13:849600. doi:10.3389/fmicb.2022.849600. 35350614 PMC8957983

[48] Ohashi Y, Hirayama A, Ishikawa T, Nakamura S, Shimizu K, Ueno Y, Tomita M, Soga T. 2008. Depiction of metabolome changes in histidine-starved Escherichia coli by CE-TOFMS. Mol Biosyst 4:135–147. doi:10.1039/b714176a. 18213407

[49] Kami K, Fujimori T, Sato H, Sato M, Yamamoto H, Ohashi Y, Sugiyama N, Ishihama Y, Onozuka H, Ochiai A, Esumi H, Soga T, Tomita M. 2013. Metabolomic profiling of lung and prostate tumor tissues by capillary electrophoresis time-of-flight mass spectrometry. Metabolomics 9:444–453. doi:10.1007/s11306-012-0452-2. 23543897 PMC3608864

[50] Hiroshima Y, Yamamoto T, Watanabe M, Baba Y, Shinohara Y. 2018. Effects of cold exposure on metabolites in brown adipose tissue of rats. Mol Genet Metab Rep 15:36–42. doi:10.1016/j.ymgmr.2018.01.005. 30023288 PMC6047462

[51] Toyoda A, Sato M, Muto M, Goto T, Miyaguchi Y, Inoue E. 2020. Metabolomic analyses of plasma and liver of mice fed with immature Citrus tumida peel. Biosci Biotechnol Biochem 84:1098–1104. doi:10.1080/09168451.2020.1719821. 32019425

[52] Iba H, Shimohata T, Kido J, Hatayama S, Uebanso T, Mawatari K, Takahashi A. 2021. Vibrio parahaemolyticus induces inflammation-associated fluid accumulation via activation of the cystic fibrosis transmembrane conductance regulator. J Med Invest 68:59–70. doi:10.2152/jmi.68.59. 33994481

[53] Watari A, Hashegawa M, Yagi K, Kondoh M. 2015. Homoharringtonine increases intestinal epithelial permeability by modulating specific claudin isoforms in Caco-2 cell monolayers. Eur J Pharm Biopharm 89:232–238. doi:10.1016/j.ejpb.2014.12.012. 25513955

