# Supplementary figures and images for: Characteristic Metabolic Changes in Skeletal Muscle Due to Vibrio vulnificus Infection in a Wound Infection Model
Source: mSystems. 2023 Mar 20;8(2):e00682-22. doi: 10.1128/msystems.00682-22 (PMC10153474; doi:10.1128/msystems.00682-22)

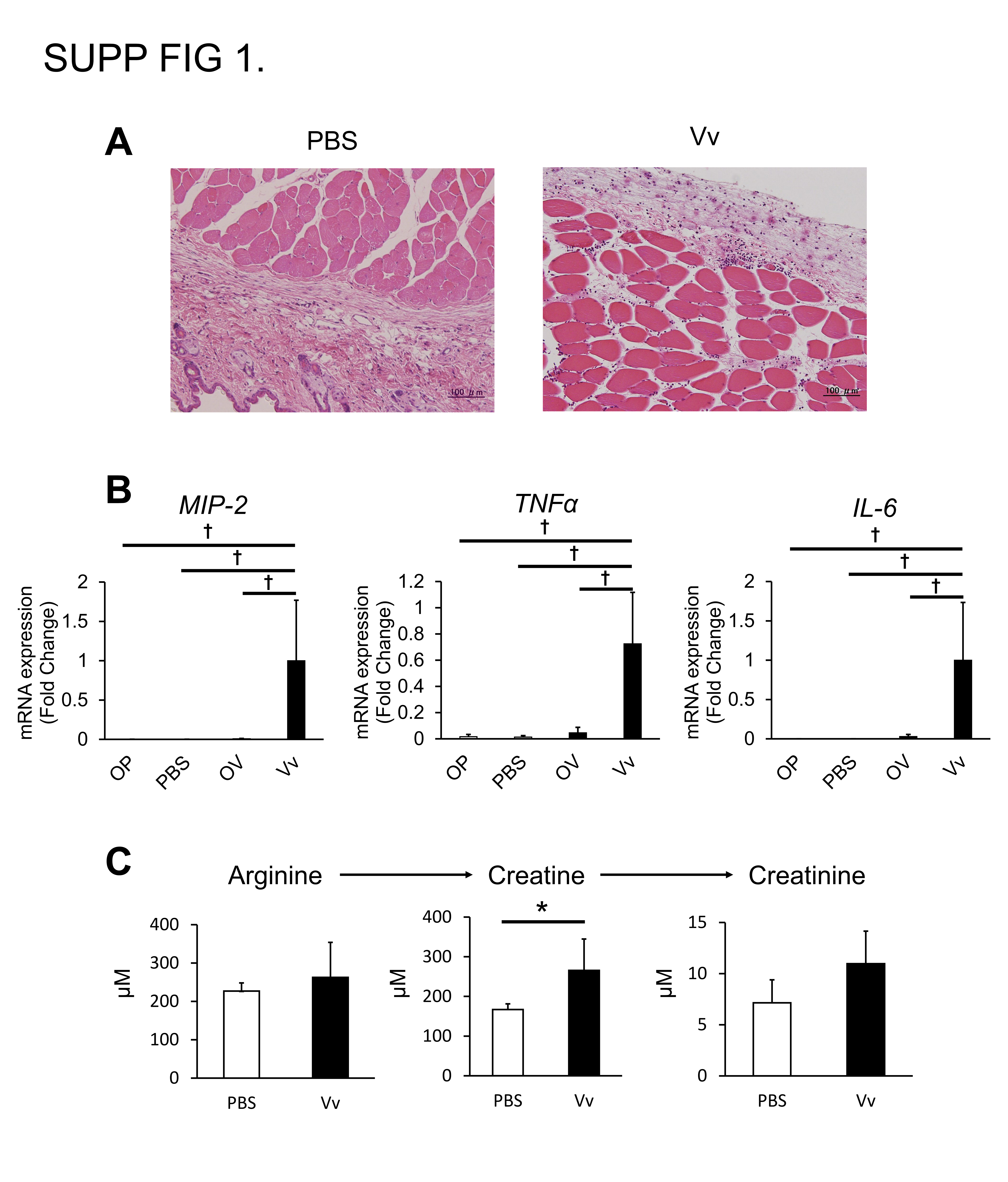

Supplement: FIG S1 [file msystems.00682-22-s0002.tif]

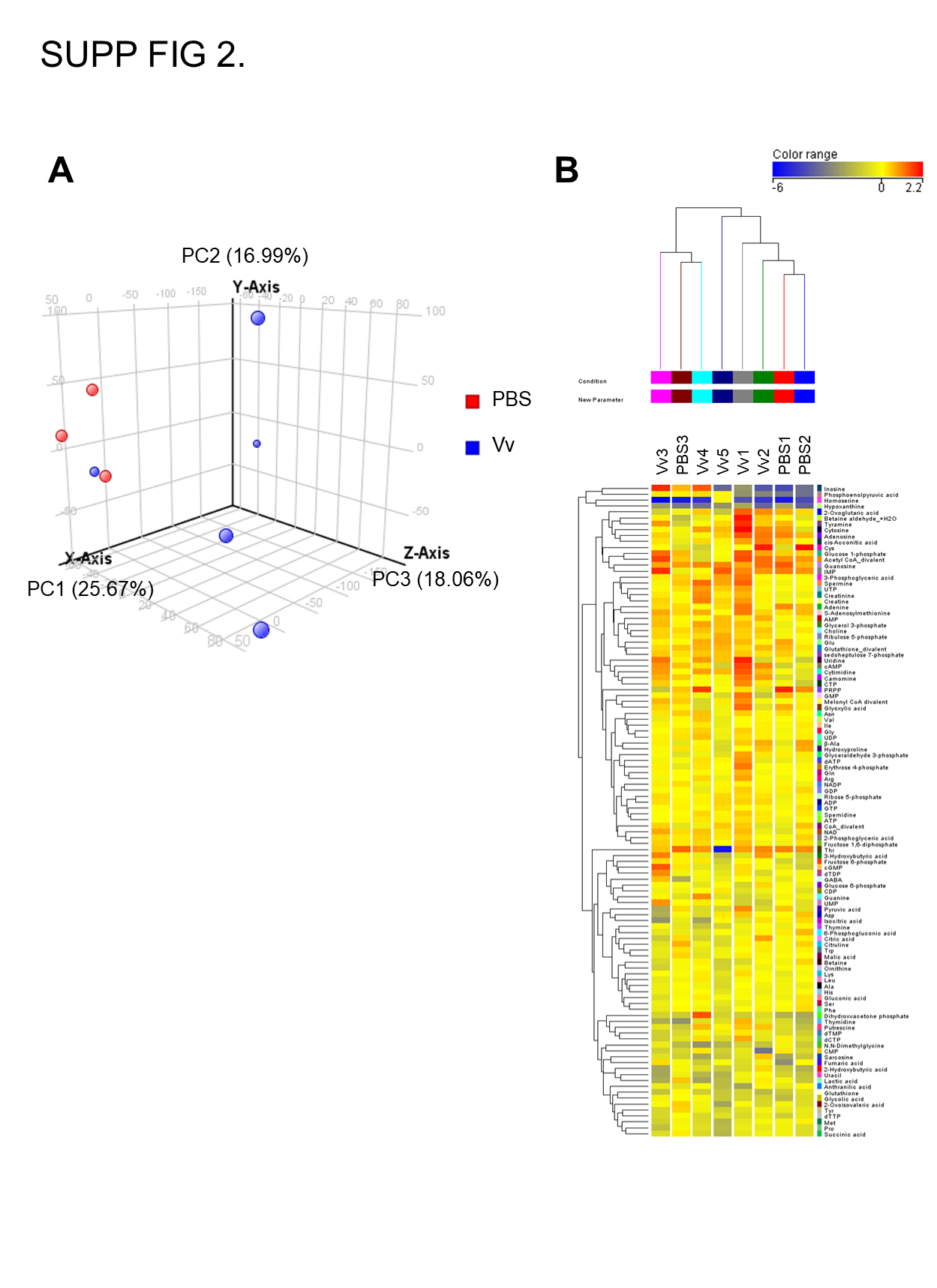

Supplement: FIG S2 [file msystems.00682-22-s0003.tif]

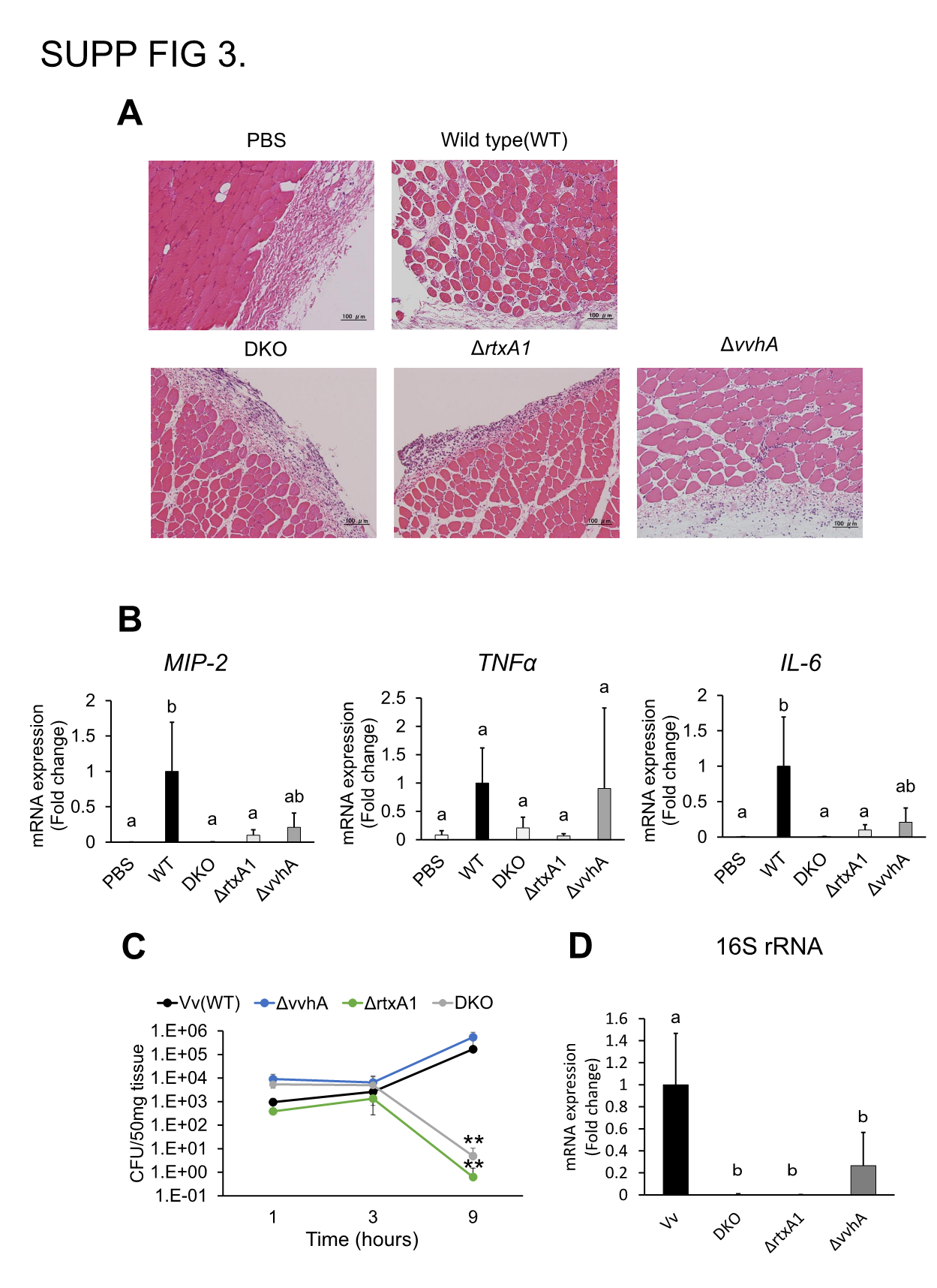

Supplement: FIG S3 [file msystems.00682-22-s0004.tif]

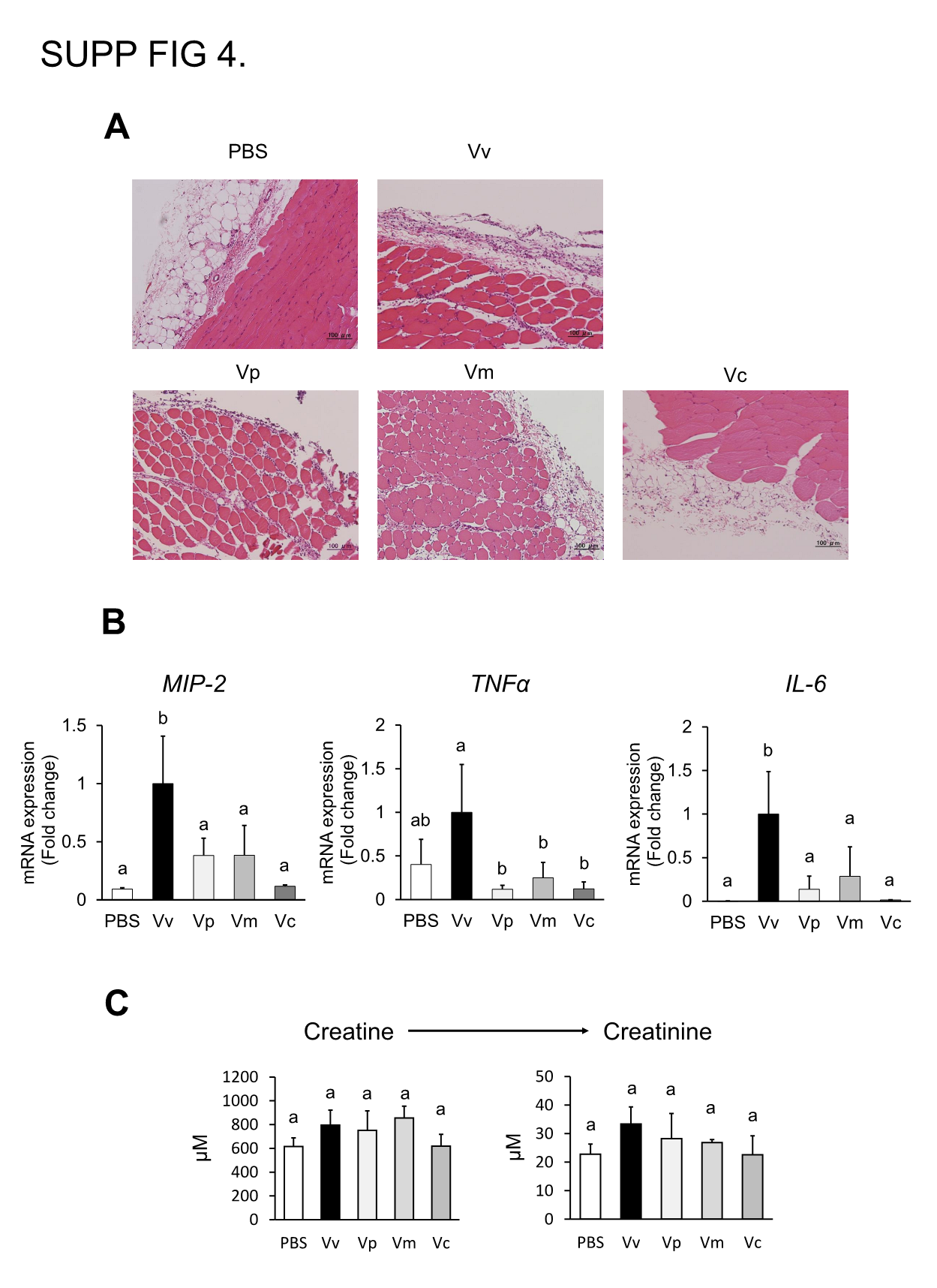

Supplement: FIG S4 [file msystems.00682-22-s0005.tif]
