# Supplementary material for: Characteristic Metabolic Changes in Skeletal Muscle Due to Vibrio vulnificus Infection in a Wound Infection Model
Source: mSystems. 2023 Mar 20;8(2):e00682-22. doi: 10.1128/msystems.00682-22 (PMC10153474; doi:10.1128/msystems.00682-22)
Supplement: TABLE S1 [file msystems.00682-22-s0001.pdf]

Supplemental Table 1.

Skeletal muscle metabolites in figure 1, 2A

m/z	MT	Component	NP			PBS			NV			Vv		
			1	2	3	1	2	3	1	2	3	1	2	3
76.0393	8.33	Gly	25431.64	19129.99	24945	21151.24	24829.52	17911.49	28700.12	25884.72	20760.64	13103.4	13502.2	10153.9
89.1073	4.72	Putrescine	878.5889	974.4329	674.1104	891.5863	1222.177	471.9321	206.5797	126.2699	325.0186	616.878	876.256	746.8
90.0550	7.31	β-Ala	755.6789	347	858.1447	468.7598	232.0839	198.3631	278.9695	1517.309	206.8659	1071.49	1297.82	1047.73
90.0550	9.02	Ala	68276.47	94161.36	150126	112384.8	96497.98	109837.9	120966.2	113191.6	113409.3	26912.5	34355.1	37104.5
90.0550	9.49	Sarcosine	34073.58	25275.21	33947.91	33927.41	29903.61	23931.32	39384.96	38026.17	35319.83	15179.6	17392.4	15218.2
104.0706	7.65	GABA	177.9596	101.5151	202.0045	327.8362	45.47328	67.62054	302.5259	88.47214	353.0992	184.861	88.4101	190.839
104.0706	10.93	N,N-Dimethylglycine	24004.7	39262.49	52040.17	23214.53	21081.95	18961.35	54148.83	23901.45	25788.91	37143.9	9408.83	30441
104.1070	6.88	Choline	498.382	573.7791	433.4212	745.2465	461.9506	763.2839	3801.269	2970.65	3232.907	13704.3	17201.5	14046.9
106.0499	10.01	Ser	7087.734	4645.448	5499.324	6406.912	5064.062	4375.059	9655.369	10260.91	7110.315	546.626	653.269	552.943
112.0505	7.26	Cytosine	1.932875	4.209882	3.929033	2.604604	3.880073	6.966672	31.48018	33.85073	35.75519	9.46613	3.41307	3.94037
113.0346	21.47	Uracil	47845.12	36304.07	49008.65	48093.11	38318.01	43740.33	20451.61	23159.18	28758.3	12696.4	13896.1	15725
114.0662	7.32	Creatinine	11979.04	8778.754	8451.173	11849.89	10618.92	6503.314	4794.95	16102.19	8101.75	1415.07	3472.62	1715.91
116.0706	10.82	Pro	4954.69	3089.763	4152.92	4562.603	4501.547	3569.02	10610.15	9245.027	8646.291	6407.72	7378.19	5536.92
118.0863	10.04	Val	2991.886	2215.016	3532.457	2976.241	2491.154	3209.51	6038.494	5605.994	5252.776	5538.77	6258.97	5077.03
118.0863	11.27	Betaine	663.1824	667.6458	844.8117	820.442	740.1795	1109.812	2086.958	1364.498	1406.102	1922.1	1886.83	1434.72
120.0655	10.09	Homoserine	53.95806	13.91365	44.69792	78.08827	21.44302	39.30677	61.22246	51.22121	52.45268	11.2158	27.9154	22.806
120.0655	10.53	Thr	5950.51	5606.551	6952.471	5672.455	6651.874	5659.094	9289.783	9619.399	7607.36	4991.24	5788.19	3933.77
120.1019	7.50	Betaine aldehyde_+H2O	6.429904	1.236088	1.60777	2.030973	2.787815	5.045514	13.02391	1.333877	5.600643	15.8464	14.7643	19.0223
122.0270	11.28	Cys	12.87955	3.788297	2.76912	7.122168	0.846428	13.95318	107.3499	8.040847	63.99968	36.5547	41.7962	22.4057
127.0502	21.37	Thymine	1133.478	933.7004	1207.633	1173.124	944.9809	1205.769	1121.334	952.1776	972.9348	856.547	870.402	1114.02
132.0655	12.00	Hydroxyproline	2166.882	1453.784	1421.289	2028.817	2235.869	1121.786	1583.547	1012.922	982.3885	625.937	489.949	386.778
132.0768	8.84	Creatine	257824.2	207203.3	303042.7	254639.8	224932.7	266531.4	308352.6	273579.4	270492.1	85751.8	77094.6	66481
132.1019	10.23	Ile	933.2401	853.6916	1252.799	1001.369	947.2826	1075.234	2895.633	2972.795	2623.25	2634.09	3151.69	2382.59
132.1019	10.33	Leu	1960.951	1635.225	2294.93	1921.162	1689.701	2016.488	4937.21	5029.156	4549.617	4486.66	5547.21	3993.11
133.0608	10.47	Asn	1043.336	902.4207	1097.182	985.8164	1097.561	966.6075	3003.962	2489.3	2008.818	318.01	593.438	463.045
133.0972	6.84	Ornithine	1813.9	1030.647	2068.939	1772.633	1046.057	1836.088	375.2308	296.717	431.6853	101.086	116.288	104.728
134.0448	11.52	Asp	1265.432	1794.835	1322.865	1289.443	2428.443	1151.198	1371.108	1503.11	1189.116	237.122	251.732	201.298
136.0618	7.58	Adenine	541.8818	290.0825	444.9449	413.0154	155.7614	363.7806	412.6432	394.0148	389.4303	261.652	241.35	178.368
137.0458	11.08	Hypoxanthine	276.7747	396.1478	204.2146	244.9641	248.4207	324.763	3845.727	5506.551	4448.491	11368.9	9569.49	8342.77
138.0550	10.61	Anthranilic acid	50.42054	44.41963	55.23279	34.70223	1.97947	44.38905	57.29578	49.28798	49.24875	48.0733	60.1997	76.5449
138.0913	8.29	Tyramine	1.422356	1.240015	0.784716	2.109257	1.602841	1.048592	3.409094	1.838218	1.004591	2.16863	1.7612	1.17194
146.1652	4.52	Spermidine	164.0307	161.211	61.95358	168.7848	199.8	64.98094	11.28884	26.7277	52.44367	6.7129	7.36039	3.55388
147.0764	10.75	Gln	24048.89	19537.57	26567.42	22470.61	24034.37	17113.49	54916.29	57576.19	36545.6	15911	16088.3	9121.64
147.1128	6.91	Lys	61206.62	27557.24	43984.56	61578.92	26143.62	38822.24	17664.87	21529.03	41577.05	10028.5	12991.4	12197.8
148.0604	10.93	Glu	18155.19	16394.28	11240.42	15111.5	24419.34	10781.18	8943.642	7832.587	5474.199	11358.1	15102.6	9943.6
150.0583	10.73	Met	2027.005	1482.561	2488.471	1867.105	1650.17	2245.028	2617.644	2310.213	2573.739	2661.57	2911.55	2811.14
152.0567	8.29	Guanine	6.936284	6.93539	6.367393	20.52543	14.91481	25.79434	27.44073	6.038927	6.657618	29.8742	3.43914	4.32322
156.0768	7.31	His	13444.35	9583.07	8035.827	13122.53	11289.27	7673.814	4268.292	15842.85	8991.485	1055.4	2965.57	1617.25
166.0863	11.09	Phe	817.9507	769.9132	1013.154	835.6547	748.5777	983.2096	3299.645	3003.032	2893.285	2795.54	3323.3	2670.71
175.1190	7.09	Arg	14480.05	5930.727	13722.87	14758.21	5070.725	10443.33	5875.285	7602.037	13187.26	4858.72	5209.14	5085.53
176.1030	11.04	Citruline	1590.977	1152.25	2622.656	1629.636	1310.633	2011.97	2083.275	2387.699	2436.653	2214.2	2443.18	2126.69
182.0812	11.34	Tyr	2507.118	1927.493	2805.272	2433.527	2007.697	2470.436	2624.415	2711.654	2360.107	2751.25	3290.72	2639.55
203.2230	4.47	Spermine	185.184	54.8907	61.37	166.6546	77.45865	65.82727	15.12849	2.235897	3.051404	6.35187	3.51148	3.95316
205.0972	11.02	Trp	293.579	314.0704	500.9354	303.9236	311.3584	351.9749	878.9838	715.0387	724.2996	877.711	916.356	742.962
227.1139	6.78	Carnosine	13181.61	9456.672	7311.965	11991.38	11399.83	6831.648	4331.291	17545.24	8428.793	1282.48	3401.97	1582.17
243.0975	21.50	Thymidine	9.14592	5.499203	17.9632	34.76546	6.238015	16.19182	30.03496	13.75667	24.31877	21.7672	23.8667	14.4412
244.0928	9.65	Cytimidine	13.26391	25.18041	15.56412	32.04601	28.72351	18.44253	335.5307	400.0944	299.3834	22.3948	9.15934	7.75887
245.0768	21.53	Uridine	269.0048	36.16899	32.46175	539.182	35.88404	67.59186	1273.148	1018.461	771.1184	266.903	328.118	131.818
268.1040	9.83	Adenosine	14.17221	4.669298	7.882446	14.06263	6.150242	11.96386	16.67889	18.87041	14.03508	9.52294	4.61586	4.47087
269.0880	19.13	Inosine	882.853	765.1659	621.5008	986.1803	654.4706	688.1614	8526.068	13111.43	13947.74	1340.15	976.04	513.33
284.0989	12.44	Guanosine	15.99306	13.92289	12.31712	18.32205	20.44778	16.77902	62.23778	87.60943	73.8506	42.8232	20.9768	1.69036
307.0833	12.13	Glutathione_divalent	581.001	849.3137	973.6559	506.0729	2493.616	579.2408	1033.466	2295.02	1181.923	625.73	770.162	608.679
308.0911	13.14	Glutathione	450.2475	851.0502	798.3395	422.9532	1725.467	476.1942	773.0847	1787.747	954.8041	466.937	627.833	439.017
399.1445	7.09	S-Adenosylmethionine	178.8878	144.7116	275.4393	199.7359	117.2834	278.0876	86.64197	70.19992	120.4569	11.972	13.0755	2.38682
75.0088	12.68	Glycolic acid	390.026	345.4676	41.04853	204.3237	449.7777	102.7899	1372.948	363.6018	894.1384	285.071	742.189	1031.62
89.0244	10.76	Lactic acid	303672.4	240948.2	333337.5	270346	263797.5	251214.1	110599.7	168535.5	124233	61797.5	84146.2	89326.9
103.0401	9.62	3-Hydroxybutyric acid	343.3784	351.8602	557.8468	373.0938	386.6047	486.2779	1088.098	415.6195	928.2965	878.809	876.353	957.245
103.0401	9.87	2-Hydroxybutyric acid	57.89424	33.69271	18.13999	23.37294	55.04623	37.40327	65.69985	38.39745	32.82932	69.8678	47.6677	66.8375
115.0037	25.02	Fumaric acid	786.0911	849.9839	923.0687	765.6163	831.5064	705.6412	622.5412	905.7484	860.2924	89.1766	59.455	123.12
115.0401	10.20	2-Oxoisovaleric acid	22.35323	19.1778	13.21339	36.26505	26.2147	62.60416	30.71278	33.62767	18.63983	17.5652	12.4407	44.4876
184.9857	19.00	2-Phosphoglyceric acid	889.6705	750.3931	549.8269	5216.107	7316.653	6173.206	288.8351	3257.059	246.2355	160.59	197.734	69.6627
191.0197	25.97	Citric acid	431.8911	499.0295	528.7963	428.9756	588.4374	537.4849	1396.973	744.0613	605.4072	1848.53	1624.27	2777.68
191.0197	28.28	Isocitric acid	23.30341	6.6591	15.57593	21.55694	15.50837	18.18349	88.29985	17.47341	18.33959	43.806	14.2803	50.818
195.0510	8.20	Gluconic acid	213.3417	610.2236	227.2335	1440.876	910.4552	1049.265	3724.806	3383.191	426.7583	2246.89	1094.31	1953.75
229.0119	10.66	Ribose 5-phosphate	371.7095	527.9158	329.3874	298.2695	434.2301	420.7159	912.4131	207.429	1034.444	103.552	94.7647	119.253
259.0224	9.83	Glucose 6-phosphate	51077.98	35409.81	43117.19	54560.13	41823.5	44341.11	41.05852	20175.24	94.69366	65.8734	93.4356	82.6276
259.0224	9.96	Fructose 6-phosphate	8272.093	5443.821	6471.464	10103.62	7630.75	8493.537	41.81255	3631.991	25.8889	47.18	30.9762	34.9174
259.0224	10.10	Glucose 1-phosphate	3961.983	2184.629	3031.202	5078.053	3720.193	4656.446	83.29574	2151.454	20.67892	525.405	1042.37	761.476
275.0174	14.67	6-Phosphogluconic acid	135.5951	185.6213	108.9524	164.0689	145.663	180.3735	24.02285	77.86079	20.42896	12.8756	35.277	41.7518
322.0446	9.66	CMP	57.53706	65.16417	74.68086	54.52751	54.83795	63.52746	34.51406	20.28392	21.62244	13.9151	6.5045	18.2104
323.0286	9.86	UMP	146.3788	203.9116	219.4777	143.1254	152.214	191.6294	67.46814	62.44766	51.75644	21.1889	7.0197	11.3545
328.0452	7.56	cAMP	216.2559	220.4411	230.0892	327.6888	329.7954	180.5778	194.5062	186.5381	75.23758	37.0198	36.3299	11.3874
338.9888	14.56	Fructose 1,6-diphosphate	19969.67	10786.47	6786.13	13480.37	11370.47	9288.177	14.31805	5509.532	17.83775	12.1159	28.5673	17.531
344.0402	7.65	cGMP	5.444608	7.81821	11.38028	23.70664	8.25305	9.394521	10.937	9.766354	4.277322	3.94062	5.36709	8.23463
347.0398	9.62	IMP	51941.19	38833.3	46663.07	43272.26	48731.29	39929.96	33406.21	25543.08	29559.94	865.62	44.418	14.5205
362.0507	9.17	GMP	86.00923	123.6318	134.6652	81.71252	118.8538	98.0908	287.977	82.89428	243.483	14.6268	6.16989	11.9077
388.9445	16.74	PRPP	41.31757	11.27613	15.487	20.6017	20.38453	14.76648	53.90129	11.25459	9.815767	23.8393	14.389	25.6811
401.0157	11.37	dTDP	17.24502	4.219303	7.518496	7.479478	7.563095	6.377839	18.3387	2.645767	4.213221	6.66759	7.43176	11.3902
402.9949	11.73	UDP	74.8122	70.53758	41.77841	60.81069	79.32892	44.81939	8.288053	76.308	10.66846	10.0285	8.60622	23.3626
403.5556	10.31	Acetyl CoA_divalent	6.804011	2.644208	2.911385	4.833413	4.666464	11.78608	10.88667	19.36945	8.619679	2.52069	2.16949	2.33856
426.0221	10.93	ADP	2255.501	1858.326	1655.299	1731.068	2256.156	1614.46	2813.268	4491.455	2759.8	1044.93	1177.52	1472.4
481.9772	12.39	CTP	40.0216	20.34764	18.26692	35.69475	46.95021	28.65759	15.04666	66.5366	13.92218	15.6064	9.43171	19.7905
505.9885	11.85	ATP	1732.215	787.5447	1353.988	1064.708	1263.393	1112.902	89.67335	8278.497	40.04723	48.8862	34.8957	45.5351
521.9834	11.55	GTP	86.83071	59.57272	64.41409	44.77417	101.5721	66.76773	30.2387	102.3199	13.55581	14.9521	15.2207	28.3068
662.1019	6.60	NAD	657.3083	344.1035	676.6213	529.8795	620.0492	474.4911	1168.82	1057.581	575.2889	78.9611	24.7344	18.2165
742.0682	9.45	NADP	91.26976	56.94333	28.70659	36.80939	15.25475	50.70786	58.50316	47.66416	32.47987	39.0671	21.9808	40.6165
117.0193	20.95	Succinic acid	1142.353	545.5113	877.5837	745.7768	510.319	586.8494	1004.759	1398.823	1005.537	4482.23	9622.2	8877.47
133.0142	21.14	Malic acid	1603.268	1900.585	1668.856	1428.333	1811.309	1330.42	1561.801	1412.333	2494.829	296.082	119.189	129.455
166.9751	20.76	Phosphoenolpyruvic acid	300.692	227.4695	192.7712	259.4449	272.3731	265.8365	94.93987	169.1232	63.21065	22.4301	7.16984	29.5145
168.9908	12.68	Dihydroxvacetone phosphate	134.2677	75.29898	69.74468	7979.19	4708.885	5699.31	33.17238	2620.223	20.14582	7.1613	13.6547	17.8604
171.0064	12.16	Glycerol 3-phosphate	2645.477	2567.176	2673.307	3121.489	2476.152	2401.201	790.2453	4938.305	628.7354	2022.5	3182.29	1584.99
173.0092	27.93	cis-Acconitic acid	8.636993	4.768275	5.665369	13.68454	4.900489	8.357007	10.10446	8.460894	5.035554	7.31548	4.99004	17.4602
184.9857	19.57	3-Phosphoglyceric acid	2754.104	2613.234	1944.894	2236.599	3499.126	2457.71	133.9663	1342.943	118.4565	76.5116	99.5107	41.2785
289.0330	9.70	sedoheptulose 7-phosphate	12.94197	27.5719	2.882993	3.253804	2.800963	2.964785	4.708595	4.454189	3.677651	31.1825	11.3543	11.8247
321.0493	9.62	dTMP	24.35899	17.74145	42.08042	5.431085	2.699663	3.081068	4.569094	3.3328	3.070809	19.0191	13.6988	20.8328
346.0558	9.30	AMP	30.36105	35.21621	66.41164	24.99557	36.62516	35.59734	451.549	511.2269	306.7288	22.1146	11.4719	15.9189
402.0109	11.51	CDP	18.91373	19.00863	12.81968	12.51558	18.99101	10.47509	7.357736	18.28765	8.590631	3.16528	4.4836	5.12987
425.5505	12.07	Melonyl CoA divalent	2.858969	3.30931	1.701095	10.19019	3.458716	5.823516	5.044777	4.908556	4.911006	5.36567	5.35489	5.03163
442.0171	10.71	GDP	26.5195	24.11379	14.3463	17.83455	26.66634	19.97247	46.23075	25.08302	26.87354	5.98493	4.88972	9.29544
465.9823	12.49	dCTP	4.96013	1.303527	1.635065	9.468155	3.777501	3.759916	3.454883	6.22517	2.433374	1.91051	2.26768	4.92268
480.9820	12.18	dTTP	5.278392	5.142217	3.712845	11.12065	3.467337	7.017433	3.044948	8.752629	2.72496	8.97184	4.49687	8.37092
482.9613	12.60	UTP	84.48693	50.59878	60.89327	54.57459	74.28314	37.77478	4.072796	51.18418	3.372054	1.01416	3.19154	4.26351
489.9936	11.79	dATP	2.487538	1.176214	1.557993	2.851735	2.704497	1.961946	2.895766	1.515887	3.036355	1.71279	2.75725	2.51905
72.9931	11.77	Glyoxylic acid	105.21	50.57642	63.17042	166.7154	87.19458	80.14955	182.4866	122.9484	77.3138	27.1976	67.82	110.893
87.0088	12.90	Pyruvic acid	479.2999	532.5973	422.8011	942.9162	477.2788	619.4293	212.5633	99.33592	147.7249	37.4729	86.0692	120.514
145.0142	21.31	2-Oxoglutaric acid	75.54907	39.08379	25.55169	54.22361	63.25491	81.17469	46.2741	48.18475	31.56101	57.001	30.1441	59.3389
168.9908	11.64	Glyceraldehyde 3-phosphate	85.14053	76.86123	63.75786	79.75676	119.1637	101.4099	64.26566	89.96609	73.04648	19.0491	24.0955	54.0333
199.0013	11.01	Erythrose 4-phosphate	4426.894	2954.871	4338.808	139.7931	91.34996	87.34749	133.5481	52.03191	47.24932	88.6617	26.1895	124.943
229.0119	11.01	Ribulose 5-phosphate	248.2804	352.6171	220.0116	226.8309	290.0405	281.0137	609.4389	138.5505	690.9488	69.1664	63.2973	71.7964
382.5503	10.69	CoA_divalent	9.512411	5.292095	4.78402	5.806446	4.390139	5.525153	3.431042	13.53674	5.873929	3.2386	3.69767	4.50354

Whole blood metabolites in figure 2B, S2

m/z	MT	Component	PBS			Vv				
			1	2	3	1	2	3	4	5
76.0393	8.33	Gly	482.6001	605.5598	637.4527	735.555	613.0019	544.7028	734.4502	446.4647
89.1073	4.72	Putrescine	2.443298	1.494952	1.745284	5.954789	5.936875	2.738877	6.441021	4.527582
90.0550	7.31	β-Ala	19.60859	51.25308	19.22333	39.69165	47.97562	31.91014	30.60213	16.44127
90.0550	9.02	Ala	561.3192	531.7896	701.9304	688.4784	552.4248	510.9482	571.7453	371.396
90.0550	9.49	Sarcosine	5.49681	9.667624	11.18298	15.27381	33.52445	14.7571	5.616296	9.62162
104.0706	7.65	GABA	4.550253	6.052526	1.728701	6.460457	5.308619	9.585694	5.328513	4.960888
104.0706	10.93	N,N-Dimethylglycine	13.40101	6.735917	5.20854	7.576167	6.021969	11.52689	2.745441	4.733048
104.1070	6.88	Choline	100.3002	138.0186	149.0943	157.935	164.7235	152.5054	171.4022	164.7889
106.0499	10.01	Ser	391.093	469.5583	400.5545	301.7752	361.3175	308.4297	374.1436	264.641
112.0505	7.26	Cytosine	1.157311	0.379134	0.522842	2.955979	1.316337	0.651275	1.121102	0.935454
113.0346	21.47	Uracil	366.6542	278.7143	573.087	593.4754	676.816	169.8826	225.1711	210.8887
114.0662	7.32	Creatinine	6.142482	6.167403	7.596288	12.32716	8.515982	8.983426	15.97533	9.049709
116.0706	10.82	Pro	143.4683	101.5437	157.1847	130.691	124.1379	63.99687	106.6873	58.48257
118.0863	10.04	Val	448.0486	483.3739	476.6851	450.6946	481.1543	338.7361	645.758	330.3492
118.0863	11.27	Betaine	152.0031	233.0142	162.1741	99.55435	191.0253	96.33048	199.1669	83.49408
120.0655	10.09	Homoserine	3.48843	8.086936	3.479324	9.398641	5.851711	2.384727	5.324882	138.1112
120.0655	10.53	Thr	302.6297	280.729	361.0199	250.9575	290.9983	198.9171	264.7081	2.931519
120.1019	7.50	Betaine aldehyde_+H2O	0.85406	0.516243	0.2799	3.518901	1.18018	0.895376	0.574091	0.87191
122.0270	11.28	Cys	1.026649	7.746831	1.042663	1.80213	7.10524	1.818647	0.804655	1.593499
127.0502	21.37	Thymine	299.5114	421.7491	267.409	325.14	383.9552	351.0752	159.0524	196.8321
132.0655	12.00	Hydroxyproline	32.73915	64.50491	45.96413	37.96998	55.73708	35.4762	42.72009	20.87266
132.0768	8.84	Creatine	144.1052	165.9089	180.1787	312.0829	186.8665	225.0998	380.2519	223.6435
132.1019	10.23	Ile	162.67	209.4685	187.7136	175.809	202.8034	196.0953	213.0391	134.3546
132.1019	10.33	Leu	229.1678	282.2459	240.5207	253.5904	294.6265	199.1652	331.5496	160.3675
133.0608	10.47	Asn	70.95596	58.97456	79.5077	78.85236	61.90804	69.03308	85.31109	49.36818
133.0972	6.84	Ornithine	101.1017	99.40951	94.46209	85.76643	87.402	49.93383	103.9533	51.32652
134.0448	11.52	Asp	52.55067	78.45776	48.96908	77.60478	52.87036	15.09647	30.91158	63.28122
136.0618	7.58	Adenine	1.92786	1.413063	1.179727	2.183419	0.820266	1.173399	0.952944	1.17926
137.0458	11.08	Hypoxanthine	3.318751	1.042099	1.525535	3.427483	1.09875	2.760434	1.350726	2.324406
138.0550	10.61	Anthranilic acid	3.827555	5.765617	3.42471	1.232355	5.876771	4.110955	5.165752	1.808199
138.0913	8.29	Tyramine	0.886177	0.566832	0.276596	2.104249	0.808291	1.091761	0.538783	0.73795
146.1652	4.52	Spermidine	72.24215	79.95485	71.74411	96.74802	80.96178	70.54363	88.98992	74.93352
147.0764	10.75	Gln	975.3091	944.8065	958.4356	1451.339	815.4309	1006.001	992.6755	871.867
147.1128	6.91	Lys	445.284	574.0054	604.5682	811.2055	617.7272	629.9969	728.2802	424.5097
148.0604	10.93	Glu	264.423	162.9479	147.1715	236.4979	164.9832	165.9867	254.7429	261.9013
150.0583	10.73	Met	100.9096	132.1726	103.2045	80.02654	104.5226	56.90408	67.32436	35.74465
152.0567	8.29	Guanine	1.593626	1.483807	1.154849	0.9035	1.152738	2.009295	3.765203	1.185039
156.0768	7.31	His	109.3815	120.4639	118.2553	116.9258	98.10155	97.87719	133.2979	83.38812
166.0863	11.09	Phe	123.5698	169.2318	141.4502	128.2347	147.7958	96.51148	111.2223	76.22001
175.1190	7.09	Arg	200.9706	232.3354	233.2271	406.152	225.9685	202.501	296.7968	181.4559
176.1030	11.04	Citruline	94.98814	138.9058	168.3939	114.5443	116.8714	66.80396	84.80359	57.35693
182.0812	11.34	Tyr	118.2571	114.8014	208.0531	98.95087	139.4445	84.2844	129.0837	78.18226
203.2230	4.47	Spermine	3.998908	4.640005	6.788638	12.60354	5.297795	4.222812	12.03335	8.343363
205.0972	11.02	Trp	62.06602	75.28716	83.03011	66.35667	78.82366	48.87279	47.62118	55.7637
227.1139	6.78	Carnosine	1.099184	1.211507	2.692215	4.562969	3.455172	3.16015	2.041371	1.708822
243.0975	21.50	Thymidine	4.736266	2.524734	1.466656	13.77842	5.828933	2.290187	5.235314	5.331991
244.0928	9.65	Cytimidine	3.190316	2.133707	3.504856	9.693639	5.151113	6.308725	6.188352	5.365434
245.0768	21.53	Uridine	16.02836	6.14242	15.46856	55.00604	7.478422	33.12935	26.31373	20.2796
268.1040	9.83	Adenosine	1.386296	1.013401	0.527475	1.881024	1.49326	0.863335	0.57297	0.888012
269.0880	19.13	Inosine	2.426647	4.635267	74.33408	8.777892	3.058905	178.6076	131.8855	3.924659
284.0989	12.44	Guanosine	1.464113	0.82825	0.984492	0.901969	1.302204	0.943949	1.122427	1.129862
307.0833	12.13	Glutathione_divalent	522.5451	409.2321	492.6869	431.951	525.5839	435.2762	540.1047	537.61
308.0911	13.14	Glutathione	110.4902	155.5474	270.1999	128.4095	85.97963	96.01422	118.2341	103.01
399.1445	7.09	S-Adenosylmethionine	7.23056	9.090076	7.692999	13.57294	7.632609	10.0553	9.773796	5.968719
75.0088	12.68	Glycolic acid	59.02641	99.25006	65.73188	63.51437	63.08601	102.2358	111.1442	51.33994
89.0244	10.76	Lactic acid	4679.579	2224.849	16213.18	7321.933	10910.37	2517.434	2163.025	3157.004
103.0401	9.62	3-Hydroxybutyric acid	183.1382	93.83568	360.4469	168.3221	498.574	586.791	196.8075	84.67597
103.0401	9.87	2-Hydroxybutyric acid	6.376452	4.386813	33.54373	13.98657	32.80096	5.95632	15.40073	10.68059
115.0037	25.02	Fumaric acid	1.538273	10.34471	9.389131	5.720132	8.011203	12.53506	4.509266	9.260669
115.0401	10.20	2-Oxoisovaleric acid	16.51881	11.74476	28.98862	18.84145	11.1131	19.20411	17.62069	4.799076
184.9857	19.00	2-Phosphoglyceric acid	1217.162	1784.087	1388.251	1649.708	1202.087	1600.276	1713.272	1453.194
191.0197	25.97	Citric acid	136.336	163.0827	255.9144	162.2038	328.8126	79.63144	90.79112	103.427
191.0197	28.28	Isocitric acid	3.72006	4.169483	12.84077	5.222132	10.23331	1.440366	1.914532	8.98056
195.0510	8.20	Gluconic acid	23.66089	35.92053	50.66838	38.24265	35.77939	23.66469	46.47299	36.18993
229.0119	10.66	Ribose 5-phosphate	44.3808	61.49188	44.20271	63.57514	60.45929	64.28307	54.50373	64.81968
259.0224	9.83	Glucose 6-phosphate	15.15994	12.5685	19.88885	9.222394	23.48095	16.83718	12.06611	17.44555
259.0224	9.96	Fructose 6-phosphate	5.944913	5.89736	5.343308	8.327589	8.058001	9.961249	9.088125	3.722206
259.0224	10.10	Glucose 1-phosphate	11.1376	10.94844	8.852347	17.94636	11.15975	16.0147	3.628001	6.246377
275.0174	14.67	6-Phosphogluconic acid	38.93684	52.52926	29.70026	27.80463	39.38978	24.42506	22.24016	34.43803
322.0446	9.66	CMP	3.716131	1.732909	1.731799	2.182317	0.32361	1.498001	3.750864	1.508358
323.0286	9.86	UMP	2.425772	1.53728	1.723393	0.995769	0.933499	4.149497	2.407714	2.186283
328.0452	7.56	cAMP	1.347559	2.15302	0.583079	3.439207	2.252612	2.140705	1.307767	0.512879
338.9888	14.56	Fructose 1,6-diphosphate	100.7919	152.2389	155.3204	194.9277	110.7538	178	169.206	130.3353
344.0402	7.65	cGMP	1.090708	0.668481	0.602221	1.139046	0.869819	2.638368	0.622565	0.537782
347.0398	9.62	IMP	11.50359	10.11762	4.461203	11.25114	10.81297	19.75321	5.042136	15.10075
362.0507	9.17	GMP	3.781001	1.908579	4.255127	6.986924	1.219872	3.237216	2.542541	1.626705
388.9445	16.74	PRPP	4.927816	2.625506	9.995681	6.441557	3.384415	2.08023	17.55835	5.192278
401.0157	11.37	dTDP	1.229913	1.169356	0.965914	1.524767	0.704076	2.577685	1.118087	0.850083
402.9949	11.73	UDP	5.535556	6.809457	8.757665	8.384864	7.557229	7.338863	10.03477	5.282652
403.5556	10.31	Acetyl CoA_divalent	1.394719	1.344947	1.013281	2.155235	1.745865	2.31132	0.387037	0.638695
426.0221	10.93	ADP	279.2132	340.7355	336.8358	423.2311	401.0973	388.8901	349.8414	415.2868
481.9772	12.39	CTP	6.300876	3.231868	10.26642	18.03339	10.21499	11.97326	8.992666	7.881138
505.9885	11.85	ATP	893.4004	1178.306	1444.884	1524.539	1417.64	1231.767	1445.61	1359.497
521.9834	11.55	GTP	26.26661	35.2405	33.90833	48.38469	31.70451	28.94298	28.87833	37.08508
662.1019	6.60	NAD	143.1365	255.7673	245.5116	314.1211	163.5084	356.4159	299.7832	247.8153
742.0682	9.45	NADP	26.40125	19.77671	42.0995	33.39592	27.86067	34.25687	24.90136	32.55652
117.0193	20.95	Succinic acid	55.55532	39.04433	196.4603	85.06299	104.2187	75.69434	70.89182	38.70359
133.0142	21.14	Malic acid	80.08595	78.75857	192.7652	138.587	111.672	84.37971	77.8069	100.9039
166.9751	20.76	Phosphoenolpyruvic acid	52.06351	45.30237	460.7338	75.4967	61.30468	510.4324	551.9303	473.2997
168.9908	12.68	Dihydroxvacetone phosphate	22.26578	18.56985	19.14453	31.62232	40.43655	35.07379	198.1352	33.47025
171.0064	12.16	Glycerol 3-phosphate	46.87784	59.32893	72.99315	96.26153	75.53782	97.31473	112.096	96.42939
173.0092	27.93	cis-Acconitic acid	3.207613	2.049829	2.282111	4.540997	5.590725	3.03137	3.271916	2.774734
184.9857	19.57	3-Phosphoglyceric acid	188.7231	181.8504	231.8115	420.0136	175.6152	207.3951	211.4026	321.4164
289.0330	9.70	sedoheptulose 7-phosphate	59.49995	37.08839	75.07585	77.14611	64.83564	76.76345	77.28841	79.43902
321.0493	9.62	dTMP	0.652071	0.532459	1.334658	1.76252	1.089175	0.69507	1.291393	1.13804
346.0558	9.30	AMP	66.60891	73.50541	51.35427	67.85039	90.47393	80.90368	56.87864	82.5267
402.0109	11.51	CDP	3.967669	2.364809	2.479809	2.139182	2.746851	2.77548	3.361683	4.046592
425.5505	12.07	Melonyl CoA divalent	3.300105	3.868894	2.08429	4.327168	1.914789	3.409482	1.159854	2.476616
442.0171	10.71	GDP	15.62768	18.27966	16.3814	20.29372	16.40005	14.08064	10.7303	16.51893
465.9823	12.49	dCTP	1.42538	0.748232	0.577081	1.986274	0.780767	0.525936	1.45885	0.631486
480.9820	12.18	dTTP	1.047342	1.617018	3.761376	1.700677	2.188114	1.606411	1.670363	1.870903
482.9613	12.60	UTP	14.35551	16.26667	19.56774	28.35826	15.0317	16.72427	29.61674	21.96204
489.9936	11.79	dATP	1.303865	1.41037	0.665454	2.494952	0.896164	1.107987	1.567196	1.386044
72.9931	11.77	Glyoxylic acid	29.08269	20.28242	20.81289	45.52133	11.40115	23.96223	10.04858	15.01386
87.0088	12.90	Pyruvic acid	51.56854	22.82171	82.87388	111.3445	30.63753	13.95082	30.66871	28.61455
145.0142	21.31	2-Oxoglutaric acid	21.99324	16.99208	14.65243	40.2197	30.52757	6.938458	21.04698	5.994401
168.9908	11.64	Glyceraldehyde 3-phosphate	13.54786	13.99591	14.03282	27.15123	17.95108	14.07686	12.63464	9.64702
199.0013	11.01	Erythrose 4-phosphate	20.42763	22.2083	15.8016	49.18322	20.69717	23.52325	25.04825	21.54479
229.0119	11.01	Ribulose 5-phosphate	99.77284	112.4664	103.6633	131.2801	127.9185	131.6949	123.496	165.7014
382.5503	10.69	CoA_divalent	0.497473	1.015049	0.81598	1.210841	0.559473	1.156993	0.684946	1.228044
